# Synthesis of Tetrapeptides Containing Dehydroalanine, Dehydrophenylalanine and Oxazole as Building Blocks for Construction of Foldamers and Bioinspired Catalysts

**DOI:** 10.3390/molecules27092611

**Published:** 2022-04-19

**Authors:** Paweł Lenartowicz, Maarten Beelen, Maciej Makowski, Weronika Wanat, Błażej Dziuk, Paweł Kafarski

**Affiliations:** 1 Faculty of Chemistry, University of Opole, ul. Oleska 48, 45-052 Opole, Poland; maarten.beelen@hotmail.com (M.B.); 2 Department of Chemistry, Odisee University of Applied Sciences, Gebroeders De Smetstraat 1, 9000 Ghent, Belgium; 3 Faculty of Chemistry, Wrocław University of Science and Technology, Wybrzeże Wyspiańskiego 27, 50-370 Wrocław, Poland; weronika.wanat@pwr.edu.pl (W.W.); blazej.dziuk@pwr.edu.pl (B.D.); 4 Faculty of Agriculture and Forestry, Warmia and Mazury University of Olsztyn, pl. Łódzki 4, 10-957 Olsztyn, Poland

**Keywords:** tetrapeptides, dehydroalanine, dehydrophenylalanine, aminomethyloxazole-4-carboxylic acid, foldamers, artzymes

## Abstract

The incorporation of dehydroamino acid or fragments of oxazole into peptide chain is accompanied by a distorted three-dimensional structure and additionally enables the introduction of non-typical side-chain substituents. Thus, such compounds could be building blocks for obtaining novel foldamers and/or artificial enzymes (artzymes). In this paper, effective synthetic procedures leading to such building blocks—tetrapeptides containing glycyldehydroalanine, glycyldehydrophenylalanine, and glycyloxazole subunits—are described. Peptides containing serine were used as substrates for their conversion into peptides containing dehydroalanine and aminomethyloxazole-4-carboxylic acid while considering possible requirements for the introduction of these fragments into long-chain peptides at the last steps of synthesis.

## 1. Introduction

The construction of bioinspired enzyme mimetics—artzymes—is an approach for obtaining catalysts that emulate enzyme activity [1,2,3,4]. Artzymes are hybrids obtained by installing an abiotic cofactor into a biomolecular scaffold. This scaffold can be any biopolymer, with proteins and oligopeptides being the most frequently used. The construction of artzymes, which are based on peptide scaffolds and usually rely on combinations of hydrophobic, α-helical fragments and non-canonical amino acids that form catalytic cavities and serve as cofactors. Such artificial enzymes are usually designed in two major ways: (i) the incorporation of cofactor-like fragments into protein or peptide backbones via late-stage functionalization [5,6,7,8,9,10] or (ii) the joining of small peptide units containing components of designed active sites followed by their submerging into helical oligopeptides (most likely foldamers) [11,12,13,14,15]. These means allow for the construction of catalysts with modes of action unseen in nature. Such helical foldamers frequently contain dehydroamino acids, which serve as units that ensure rigid structures and/or the functionalization of the side chain of the peptide [16,17,18,19,20,21].

Dehydropeptides are a class of compounds containing at least one residue of an α,β-dehydroamino acid, and they are characterized by well-defined and rigid architectures [16,17,18,19,20,21,22,23,24,25]. Because of their enhanced stability in body fluids and similarity to natural peptides, they are good peptide mimics and considered an interesting and biocompatible class of physiologically active substances [26]. Additionally, their double bonds, especially those deriving from dehydroalanine, are reactive and thus easy to functionalize by introducing various substituents of complex structures [21,27,28,29,30,31,32,33,34,35]. In consequence, dehydroamino acids not only cause the rigidification of peptidyl structures but also enable the late-stage functionalization of the peptide via the introduction of the chemical entities that could form the mimetic of active site and/or introduce specific cofactor-like fragments. This is how artzymes are obtained

Oxazoles comprise an important class of five-membered heterocyclic compounds with numerous applications ranging from medicine to agrochemistry [36,37,38]. The presence of an oxazole moiety in peptide chain confers stability and a specific electronic distribution [39,40,41,42], ensuring that fragments containing these entities are good scaffolds for helical foldamer design.

The structural features of both classes of these peptide mimetics could be used to control and modify the structure, conformation, and function of a target foldamer or artzyme. For this purpose, there is a need for versatile methods for the preparation of short peptides containing multiple dehydroamino acids or dehydroamino acid–oxazole hybrids. Such methods for the synthesis of peptides containing single dehydroamino acids or oxazoles have been well-elaborated [43,44,45,46,47,48,49,50,51,52,53,54]. However, the introduction of two or more dehydroamino acids in close vicinity to each other remains a challenge [55]. In addition to its standard influence on peptidyl structures, the appearance of two neighboring double bonds enables the stapling of a peptide in order to stabilize an α-helical secondary structure [56]. Due to low availability, peptides containing multiple dehydroamino acids have not been utilized for foldamer or artzyme synthesis so far.

**Scheme 1:**
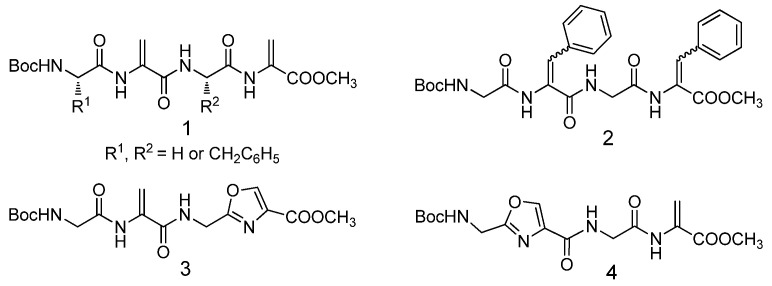
Structures of the tetrapeptide mimetics.

In our research, we studied the synthesis of tetrapeptide mimetics (Scheme 1) bearing two dehydroalanine or dehydrophenylalanine residues (compounds **1** and **2**) or one dehydroalanine residue and oxazole fragment (compounds **3** and **4**).

## 2. Results

### 2.1. Tetrapeptides Containing Two Dehydroalanine Units

Due to the low reactivity of the carboxyl group of dehydroalanine towards activating agents in coupling reactions and the instability of its totally or partially deprotected derivatives, most dehydroamino acid derivatives have limited application in peptide synthesis [45,46,47,48,49]. Thus, the majority of the procedures for their synthesis are based on the dehydration of β-hydroxy moiety of serine, the reactions that mimic their biosynthesis and introduce double bonds in the last steps of synthesis [43,44,47,48,49]. Unfortunately, the active intermediates are reactive, which can cause unwanted side reactions and present low to moderate yields of desired peptides [43,44]. Inspired by a mild procedure employed previously [43,44,48], we decided to obtain properly protected Gly-Ser-Gly-Ser and Phe-Ser-Phe-Ser without blocking serine hydroxyl moieties during the coupling steps and then introduce double bonds in one step at the end of synthetic pathway.

**Scheme 2:**
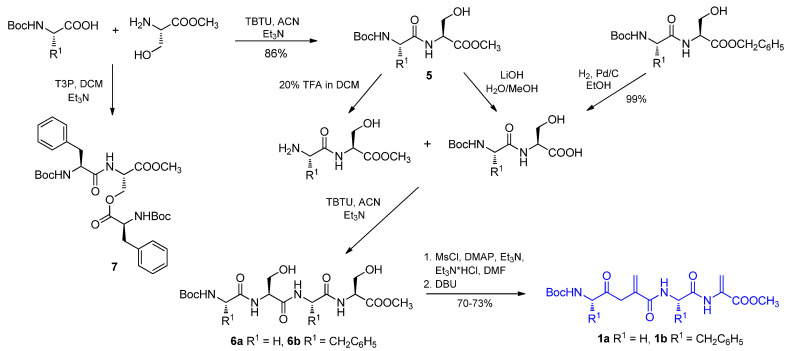
Synthetic procedure leading to compounds **1a** and **1b**.

Our method of obtaining these tetratpeptides is shown in Scheme 2. Starting from suitable dipeptides (compound **5**) obtained by coupling Boc-glycine or Boc-phenylalanine with serine methyl ester through the standard TBTU [2-(1H-benzotriazole-1-yl)-1,1,3,3-tetramethylaminium tetrafluoroborate] procedure failed because the selective alkaline hydrolysis of the methyl esters of compound **5** was accompanied with the racemization of dipeptide **5** (see Figure S1 in the Supplementary Materials). The use of a benzyl ester of serine (a protection that can be readily removed with hydrogenolysis) provided dipeptides **6a** and **6b** as pure enantiomers (see Figure S2 in the Supplementary Materials). Surprisingly, the use of another procedure in which Boc-phenylalanine was used as a substrate and T3P (propylphosphonic anhydride) was used as a coupling agent nearly exclusively yielded product **7** due to the acylation of the hydroxyl moiety of serine (see Figure S3 in the Supplementary Materials).

The procedure affording tetrapeptides **1a** and **1b** was carried out in DMF via the reaction of peptides **6a** and **6b** with methanesulfonyl chloride in the presence of triethylamine, followed by the action of an excess of DBU (Scheme 2). This reaction was tricky and proceeded faster when carried out in the presence of dimethylaminopyridine, which catalyzed the formation of active sulphonic esters of both serines. Additionally, the reaction yield increased from 57 to 70% when one equivalent of triethylamine hydrochloride was present in the reaction mixture (see Figure S4 in the Supplementary Materials). The rationalization of this complex procedure is difficult. It is also worth mentioning that the prolongation of the reaction time or increases in its temperature decreased the yield and led to polymeric products. 

**Scheme 3:**
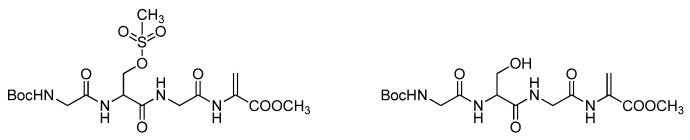
Major side-products of the dehydration reaction of **6a**, as determined by mass spectrometry.

Chromatographic fractions containing side-products of the dehydration reaction of **6a** performed without the presence of triethylamine hydrochloride were studied with high-resolution mass spectrometry (see Supplementary Materials) supplemented by NMR. The results demonstrated the presence of two major compounds, namely partially activated and partially dehydrated substrates alongside unreacted substrate and the desired product (Scheme 3). This indicates that the dehydration of serine present in the middle chain of the peptide was more difficult than the dehydration of the serine at the C-terminal end. This could have been a result of the lower acidity of the hydrogen bonded to the α carbon atom. As mentioned above, the addition of triethylamine hydrochloride to the reaction mixture significantly increased the yield and facilitated the separation of pure product from the reaction mixture.

**Scheme 4:**
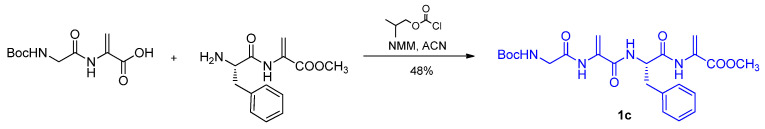
Synthesis of **1c** by coupling reaction.

Then, we compared the potency of the above-described method for the preparation of tetrapeptides **1a** and **1b** with the standard coupling of two suitably protected dehydropeptides. There is only one combination of peptidic substrates that leads to the desired products, namely the coupling of dipeptides containing dehydroalanine residues at the C-terminal end, since the activation of the carboxyl group of dehydroalanine with a variety of coupling agents is not effective. Based on our experience, we used a mixed anhydride procedure. As an example of this reaction, the Boc-Gly-ΔAla-l-Phe-ΔAla-OMe peptide (compound **1c**) was prepared using an isobutyl chloroformate reagent in the presence of *N*-methylmorpholine as a base (Scheme 4). Compound **1c** was obtained with a moderate yield of 48%. In addition to the difficulties with the coupling step, the preparation of the substrate could also be a limiting step because during deprotection, polymerization may occur as a side reaction during the reaction of Boc-XX-ΔAla-OMe with trifluoroacetic acid, thus reducing overall yield. Considering all the above inconveniences, the dehydration of β-hydroxy amino acid seems to be a suitable procedure for the synthesis of tetrapeptides **1**.

### 2.2. Tetrapeptides Containing Two Dehydrophenylalanine Units

Contrary to the tetrapeptides containing dehydroalanine units, the expanding dehydroamino acid side chain (e.g., via the presence of phenyl group in the case of dehydrophenylalanine) allows for the possibility of the formation of geometrical isomers. Therefore, dehydrophenylalanine may occur as a *Z* or *E* isomer, with the first one being of higher thermodynamic stability. The presence of two dehydrophenylalanine units in the peptide sequence leads to a combination of the four isomers that exhibit different conformational properties. Moreover, in the case of peptides containing (*E*)-isomers, difficulties result from the easy isomerization of *E*-dehydrophenylalanine when the starting substrate contains this amino acid as a C-terminal component. This leads to the obtainment of mixtures of isomers, whose resolution is usually the limiting step of the synthesis. 

**Scheme 5:**
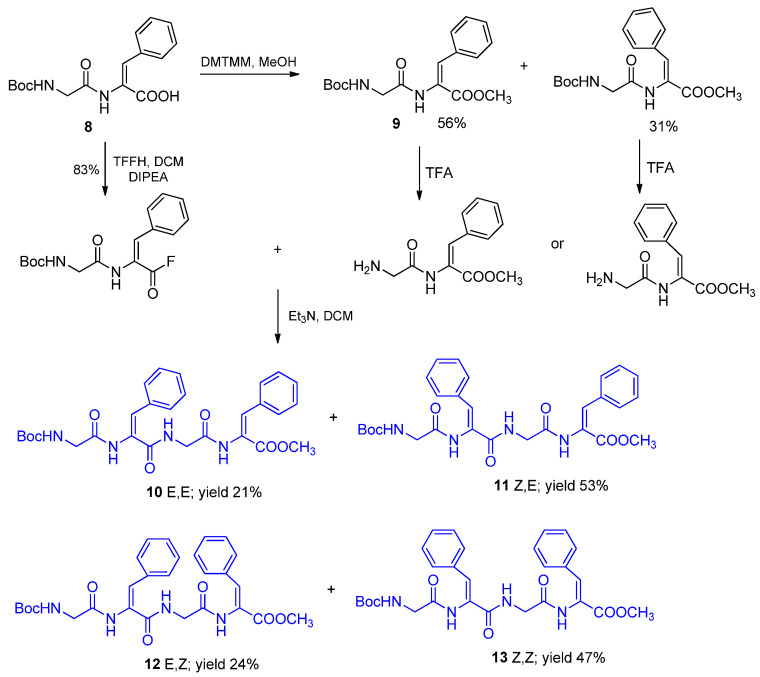
Synthesis of four isomers of glycyl-dehydrophenylalanyl-glycyl-dehydrophenylalanine.

In this part of the study, the preparation of four tetrapeptide isomers (compounds **10**–**13**) using Boc-Gly-^E^∆Phe fluoride as the acylating agent in the critical step of the synthesis is elaborated. The synthetic procedure is outlined in Scheme 5 and began with readily available Boc-glycyl-(*E*)-dehydrophenylalanine (Boc-Gly-^E^∆Phe, compound **8**) as the substrate [50,51]. Its conversion into the second dipeptidyl substrate Gly-^E^∆Phe-OMe (compound **9**) achieved via esterification with methanol by using DMTMM [4-(4,6-dimethoxy-1,3,5-triazin-2-yl)-4-methylmorpholinium chloride] was accompanied with the isomerization of dehydrophenylalanine and required the chromatographic separation of the resulting isomers. The conversion of Boc-Gly-^E^∆Phe into its fluoride by using TFFH (tetramethylfluoroformamidinium hexafluorophosphate) and its use in the acylation of Gly-^E^∆Phe-OMe or Gly-^Z^∆Phe-OMe resulted in: (i) a non-equimolar mixture of Boc-Gly-^E^∆Phe-Gly-^E^∆Phe-OMe (compound **10**) and Boc-Gly-^Z^∆Phe-Gly-^E^∆Phe-OMe (compound **11**) in the first reaction; (ii) a non-equimolar mixture of Boc-Gly-^E^∆-Phe-Gly-^Z^∆-Phe-OMe (compound **12**) and Boc-Gly-^Z^∆Phe-Gly-^Z^∆Phe-OMe (compound **13**) in the second reaction. The isomers obtained in both reactions were once more separated using column chromatography with the relative isomer ratios of 1:2.5 for E,E/Z,E and 1:2.0 for E,Z/Z,Z. 

In studying the synthetic approach presented in this section, we found that using one isomer, namely (Boc-glycyl-(*E*)-dehydrophenylalanine), as the starting substrate enabled us to obtain all four isomers of the desired tetrapeptide alternately containing protein and dehydroamino acids. It should be mentioned that the application of the Boc-Gly-^Z^∆Phe fluoride seemed to be most beneficial because of its lack of isomerization, but this approach enabled us to obtain only *Z*,*E* or *Z*,*Z* isomers. Therefore, the optimal synthetic pathway can be chosen depending on the desired output. 

### 2.3. Hybrid Tetrapeptides Containing Dehydroalanine and Oxazole Units

**Scheme 6:**
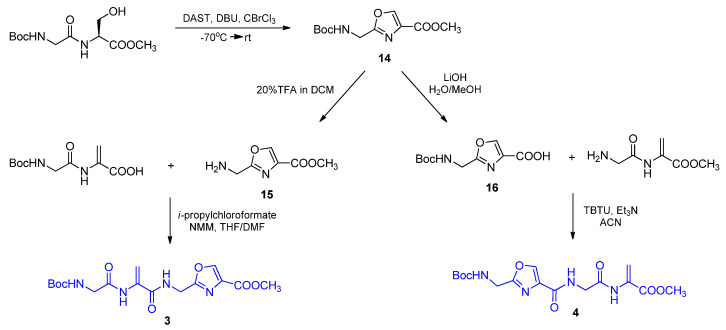
Syntheses of tetrapeptides containing dehydroalanine and oxazole units.

The methyl ester of Boc-glycyl-l-serine was used as the staring agent. Its one-pot cyclization and oxidative aromatization (Scheme 6) yielded methyl 2-(1-(tert-butoxycarbonylamino)methyl)oxazole-4-carboxylate (compound **14**) [52,53]. The selective removal of its protecting groups afforded substrates suitable for the synthesis of compounds **3** and **4** (Scheme 5). 

**Figure 1:**
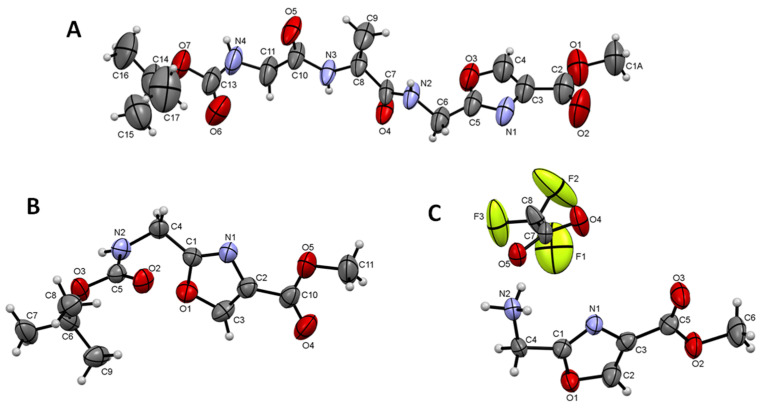
Crystal structures of: (**A**) final tetrapeptide **3** and its substrates (**B** and **C**, respectively) **14** and **15** × TFA. Displacement ellipsoids are drawn at the 50% probability level.

Thus, the coupling of Boc-glycyl-dehydroalanine with methyl 2-(aminomethyl)oxazole-4-carboxylate (compound **15**) afforded peptide **3**. In order to obtain a tetrapeptide of the reversed positioning of dehydroalanine and oxazole 2-(1-(tert-butoxycarbonylamino)methyl)oxazole-4-carboxylic acid (compound **16**) was coupled with methyl glycyl-dehydroalaninate to yield the desired tetrapeptide **4**. The structures of compounds **3, 14**, and **15** were confirmed with X-ray analysis at 293 K (Figure 1). The structural data obtained for compound **14** agreed well with previously reported results obtained at 213 K [54].

## 3. Conclusions

In this study, simple and effective methods for the synthesis of tetrapeptides composed of two glycyl-dehydroalanine, glycyl-dehydrophenylalanine, and glycyloxazole subunits are described (Table 1). The advantages and problems that can arise during the preparation of the desired dehydropeptides are summarized in Table S1 in the Supplementary Materials. These syntheses yielded tetrapeptides containing two rigidizing and reactive fragments in close vicinity, and they could be considered preliminary steps in the preparation of entities useful for the preparation of foldamers and/or artzymes, because dehydroamino acids and oxazoles enforce rigid conformations of the whole peptide structure [24,40,41,57,58,59] and could be also used for the attachment of functional groups (synthetic co-factors) via the addition of nucleophiles to their double bonds.

## 4. Experimental Section 

### 4.1. Materials and Methods

Acetonitrile (ACN), ethyl acetate (EtOAc) dichloromethane (DCM), and chloroform (CHL) were refluxed over P_2_O_5_ and distilled. *N*,*N*-Dimethylformamide (DMF) extra dry (Acros Organic), methanol (MeOH), and reagents were purchased from commercial suppliers, were of analytical grade, and were used without further purification. The reaction progress was monitored with thin layer chromatography on Merck 60 F_254_ silica plates with a fluorescent indicator. The spots were visualized with a potassium permanganate solution or a chlorine/*o*-tolidine reaction. The compounds were purified with flash chromatography using silica gel 60 (0.040–0.063 mm) from Merck. The ^1^H and ^13^C NMR spectroscopic experiments were performed on a Bruker Ultrashield Spectrometer (Bruker, Rheinstetten, Germany) operating at 400 (^1^H) MHz and 101 MHz (^13^C). The samples were prepared in d6-DMSO (99.8 at. %D) or d6-acetone (99.9 at. %D) and analyzed at 297 K. Chemical shifts are reported in ppm relative to TMS or solvent signal, while coupling constants are reported in Hz. High-resolution mass spectra (HRMS) were recorded with a Waters LCT Premier XE mass spectrometer equipped with an ESI source in the positive ion mode (Waters, Milford, MA, USA). 

### 4.2. Syntheses

#### 4.2.1. Substrates

All the fully protected dipeptides containing C-terminal dehydroamino acids were synthesized by the described procedures [30,31,45,46,50,51]. The removal of their protecting groups and the synthesis of components for the generation of serine-containing dipeptides was performed with standard peptide chemistry procedures. Methyl 2-(1-(*tert*-butoxycarbonylamino)methyl)oxazole-4-carboxylate (compound **14**) was obtained by following a previously described procedure [52,53]. Detailed descriptions of the procedures for the preparation of the substrates are provided in the Supplementary Materials. 

#### 4.2.2. Dehydratation of Tetrapeptides **6a** or **6b**—An Optimized Procedure

We placed 0.168 g (0.4 mmol, 1 eq) of Boc-Gly-Ser-Gly-Ser-OMe (**6a**) or 0.240 g (0.4 mmol, 1 eq) of Boc-l-Phe-l-Ser-l-Phe-l-Ser-OMe (**6b**), 0.055 g of triethylamine hydrochloride (0.400 mmol, 1 eq), and 0.0024 g of DMAP (0.02 mmol, 0.05 eq) into a flask and flushed it with dry nitrogen. Then, DMF extra dry (5 mL) was slowly added to dissolve the substrates. The reaction mixture was placed in an ice bath (−10 °C); then, 0.080 mL (1.04 mmol, 2.6 eq) of mesyl chloride was added, immediately followed by the dropwise addition of 0.144 mL (1.04 mmol, 2.6 eq) of triethylamine over a time span of 15 min. The mixture was stirred for an additional 60 min. Then, 0.347 mL (2.32 mmol, 5.8 eq) of DBU was added dropwise at −10 °C. The mixture was warmed to room temperature and stirred for a further 90 min. The volatile components were removed under reduced pressure, and the components were separated by means of flash column chromatography. The chromatographic conditions for appropriate peptides are given below.

▪ Methyl Boc-glycyl-dehydroalanyl-glycyl-dehydroalaninate (Compound **1a**)

The first five fractions were eluted with a mixture of EtOAc in hexane using an increasing gradient from 50% to pure EtOAc; the next fractions were eluted with a gradient of methanol in EtOAc (from 0 to 10%). The fractions were collected, and solvents were evaporated under reduced pressure. We obtained 0.108 g (0.281 mmol, 70% yield) of the product as a white solid. Mp = 80–82 °C; Rf = 0.56 (5% MeOH in EtOAc). 

^1^H NMR (400 MHz, DMSO) δ (ppm): 9.38 (s, 1H), 9.09 (s, 1H), 8.80 (t, *J* = 6.0 Hz, 1H), 7.26 (t, *J* = 6.0 Hz, 1H), 6.22 (s, 1H), 6.19 (s, 1H), 5.71 (s, 1H), 5.54 (s, 1H), 3.95 (d, *J* = 6.0 Hz, 2H), 3.76 (s, 3H), 3.66 (d, *J* = 6.0 Hz, 2H), 1.38 (s, 9H).

^13^C NMR (101 MHz, DMSO) δ (ppm): 169.08, 168.35, 164.15, 163.80, 155.95, 134.60, 132.40, 109.28, 102.77, 78.43, 52.75, 44.26, 43.08, 28.18. 

HRMS (ESI): m/z calculated for C_16_H_24_N_4_O_7_ [M + Na]^+^: 407.1543; found: 407.1547.

▪ Methyl Boc-l-phenylalanyl-dehydroalanyl-l-phenylalanyl-dehydroalaninate (Compound **1b**)

The reaction mixture was separated using an increasing gradient of EtOAc in hexane (from 20 to 100%) and then a gradient of MeOH in EtOAc (from 0 to 5%). We obtained 0.165 g (0.29 mmol, 73% yield) of the product as a white solid; Rf = 0.55 (5% MeOH in chloroform). 

^1^H NMR (400 MHz, DMSO) δ (ppm): 9.60 (s, 1H), 9.11 (s, 1H), 8.75 (d, J = 8.1 Hz, 1H), 7.40–7.14 (m, 11H), 6.23 (s, 1H), 6.21 (s, 1H), 5.75 (s, 1H), 5.52 (s, 1H), 4.85–4.75 (m, 1H), 4.29–4.16 (m, 1H), 3.77 (s, 3H), 3.14–2.92 (m, 3H), 2.70 (dd, J = 13.8, 11.1 Hz, 1H), 1.26 (s, 9H).

^13^C NMR (101 MHz, DMSO) δ (ppm): 171.25, 171.01, 163.97, 163.81, 155.41, 138.10, 137.85, 134.32, 132.52, 129.23, 129.18, 128.11, 128.08, 126.42, 126.26, 109.97, 78.43, 56.67, 55.39, 52.75, 36.63, 36.46, 28.08. The signal derived from one carbon atom of the ΔAla residue is not visible on the NMR spectrum due to high background noise. 

HRMS (ESI): m/z calculated for C_30_H_36_N_4_O_7_ [M + Na]^+^: 587.2482; found: 587.2408.

#### 4.2.3. Synthesis of Tetrapeptides and Their Oxazole Counterparts by Coupling Reaction

▪ Methyl Boc-glycyl-dehydroalanyl-l-phenylalanyl-dehydroalaninate (Compound **1c**)

We dissolved 0.144 g (0.59 mmol, 1 eq) of Boc-Gly-ΔAla-OH in dry ACN (5 mL), and then we added 0.068 mL (0.62 mmol, 1.05 eq) of *N*-methylmorpholine. The mixture was cooled to –15 °C, and 0.080 mL (0.62 mmol, 1.05 eq) of isobutyl chloroformate was added dropwise. After 15 min, the solution comprising 0.235 g (0.65 mmol, 1.1 eq) of L-Phe-ΔAla-OMe*TFA and 0.075 mL (0.68 mmol, 1.15 eq) of *N*-methylmorpholine in a mixture of ACN (5 mL) and DMF (0.5 mL) was added dropwise to the reaction mixture. The reaction was stirred at room temperature overnight. The volatile components were removed under reduced pressure, and oil residue was dissolved in EtOAc (60 mL). The organic phase was washed with 1M HCl (3 × 5 mL), a saturated solution of KHCO_3_ (3x5 mL), and brine (3 × 5 mL) before being dried over anhydrous Na_2_SO_4_. The solvent evaporation under reduced pressure yielded an oily residue that was purified by means of flash column chromatography using an increasing gradient of EtOAc in hexane (from 20 to 100%) as the eluent. We obtained 0.134 g (0.28 mmol, 48% yield) of the product, which was crystallized from the EtOAc/hexane mixture, as a white solid.

^1^H NMR (400 MHz, DMSO) δ (ppm): 9.57 (s, 1H), 8.96 (s, 1H), 8.73 (d, *J* = 8.1 Hz, 1H), 7.42–7.11 (m, 6H), 6.22 (s, 1H), 6.15 (s, 1H), 5.74 (s, 1H), 5.49 (s, 1H), 4.84–4.74 (m, 1H), 3.77 (s, 3H), 3.61 (d, *J* = 5.9 Hz, 2H), 3.09 (dd, *J* = 13.6, 3.9 Hz, 1H), 2.97 (dd, *J* = 13.6, 11.2 Hz, 1H), 1.36 (s, 9H).

^13^C NMR (101 MHz, DMSO) δ (ppm): 170.95, 168.99, 163.96, 163.80, 155.90, 137.87, 134.33, 132.53, 129.23, 128.11, 126.42, 109.97, 102.85, 78.39, 55.35, 52.74, 44.20, 36.44, 28.15.

HRMS (ESI): m/z calculated for C_23_H_30_N_4_O_7_ [M + Na]^+^: 497.2012; found: 497.2021.

▪ Methyl 2-(1-(tert-butoxycarbonyl-glycyl-dehydroalanyl)methyl)oxazole-4-carboxylate (Compound **3**)

We dissolved 0.049 g (0.20 mmol, 1 eq) of Boc-Gly-ΔAla-OH in dry THF (2 mL) under an argon atmosphere and added 0.022 mL (0.20 mmol, 1 eq) of *N*-methylmorpholine. The mixture was cooled to −15 °C, and 0.027 mL (0.21 mmol, 1.05 eq) of isobutyl chloroformate was added dropwise. After 30 min, the solution comprising 0.054 g (0.20 mmol, 1 eq) of Gly-Ozl-COOMe*TFA and 0.022 mL (0.20 mmol, 1 eq) of *N*-methylmorpholine in dry THF (2 mL) with one drop of DMF was added to the reaction mixture. The reaction was stirred at room temperature overnight. The volatile components were removed under reduced pressure, and the brown residue was dissolved in water and extracted with EtOAc (3 × 5 mL). The organic phase was washed with 1M HCl (3 × 5 mL), a saturated solution of KHCO_3_ (3 × 5 mL), and brine (3 × 5 mL) before being dried over anhydrous Na_2_SO_4_. Under reduced pressure, the solvent evaporation yielded oily residue, which was purified by means of flash column chromatography using a mixture of 5% MeOH in CHL as the eluent. Then, 0.043 g (0.11 mmol, 55% yield) of the product was crystallized from MeOH at −20 °C and obtained as a white solid. The structure of the product was confirmed with crystallographic analysis. 

^1^H NMR (400 MHz, DMSO) δ (ppm): 9.18 (t, *J* = 5.7 Hz, 1H), 9.06 (s, 1H), 8.80 (s, 1H), 7.25 (t, *J* = 6.0 Hz), 6.23 (s, 1H), 5.56 (s, 1H), 4.51 (d, *J* = 5.7 Hz), 3.79 (s, 1H), 3.65 (d, *J* = 6.0 Hz), 1.37 (s, 1H). 

^13^C NMR (151 MHz, Acetone) δ (ppm): 169.67, 164.88, 162.76, 161.95, 156.98, 145.72, 135.22, 134.14, 102.12, 79.79, 52.01, 45.76, 37.49, 28.54.

HRMS (ESI): m/z calculated for C_16_H_22_N_4_O_7_ [M + Na]^+^: 405.1386; found: 405.1396.

▪ Methyl 2-(1-(tert-butoxycarbonylamino)methyl)oxazole-4-glycyl-dehydroalaninate (Compound **4**)

We dissolved 0.062 g (0.25 mmol, 1 eq) of Boc-Gly-Ozl-COOH and 0.036 mL (0.26 mmol, 1.05 eq) of Et_3_N in ACN (10 mL), placed them in an ice bath (−10 °C), and then added 0.080 g (0.25 mmol, 1 eq) of TBTU. After 10 min, a solution comprising 0.079 g (0.30 mmol, 1.2 eq) of Gly-ΔAla-OMe*TFA and 0.045 mL (0.33 mmol) of Et_3_N in ACN (10 mL) was added dropwise to the reaction mixture. The Boc deprotected peptide was obtained directly before the reaction from Boc-Gly-ΔAla-OMe. The melted ice bath was removed, and the reaction was continued at room temperature for 12 h. The volatile components were removed under reduced pressure, and the solid residue was dissolved in EtOAc (100 mL). The organic phase was washed with 1M HCl (3 × 5 mL), a saturated solution of KHCO_3_ (3 × 5 mL), and brine (3 × 5 mL) before being dried over anhydrous MgSO_4_. We obtained 0.063 g (0.16 mmol, 66% yield) of the product, which crystallized from ethanol/hexane mixture, as a white solid; Rf = 0.57 (5% MeOH in EtOAc).

^1^H NMR (400 MHz, DMSO) δ (ppm): 9.40 (s, 1H), 8.58 (s, 1H), 8.45 (t, *J* = 6.0 Hz, 1H), 7.59 (t, *J* = 6.0 Hz, 1H), 6.25 (s, 1H), 5.72 (s, 1H), 4.29 (d, *J* = 6.0 Hz, 2H), 4.01 (d, *J* = 6.0 Hz, 2H), 3.76 (s, 3H), 1.39 (s, 9H).

^13^C NMR (101 MHz, DMSO) δ (ppm): 168.41, 163.79, 161.95, 160.35, 155.58, 142.14, 135.62, 132.30, 109.07, 78.55, 52.77, 42.62, 37.36, 28.18.

HRMS (ESI): m/z calculated for C_16_H_22_N_4_O_7_ [M + Na]^+^: 405.1386; found: 405.1396.

▪ Methyl Boc-glycyl-l-serinyl-glycyl-l-serinate (Compound **6a**)

We dissolved 0.463 g (1.77 mmol, 1 eq, obtained from benzyl ester) of Boc-Gly-l-Ser-OH, 0.451 g (2.12 mmol, 1.2 eq) of Gly-l-Ser-OMe×HCl, and 0.6 mL (4.3 mmol) of Et_3_N in ACN (20 mL). The reaction mixture was stirred and placed in the ice bath (−10 °C), and then 0.568 g (1.77 mmol, 1 eq) of TBTU was added in portions over a time span of 1 h. The ice bath was removed, and the reaction mixture was stirred at room temperature for 12 h. Then, the volatile components were removed under reduced pressure, and the reaction mixture was separated by means of flash column chromatography. The product was eluted using an increasing gradient of MeOH in the EtOAc:DCM (1:1 *v/v*) mixture from 0 to 25%. We obtained 0.434 g (1.04 mmol, 59% yield) of the product as a dense oil; Rf = 0.51 (30% MeOH in EtOAc: DCM (1:1 *v/v*) mixture).

^1^H NMR (400 MHz, DMSO) δ (ppm): 8.21 (t, *J* = 5.8 Hz, 1H), 8.11 (d, *J* = 7.6 Hz, 1H), 7.93 (d, *J* = 7.3 Hz, 1H), 7.00 (t, *J* = 5.9 Hz, 1H), 5.08 (t, *J* = 5.8 Hz, 1H), 4.99 (t, *J* = 5.6 Hz, 1H), 4.36–4.30 (m, 1H), 4.30–4.22 (m, 1H), 3.77 (d, J = 5.7 Hz, 2H), 3.72–3.50 and 3.62 (m and s overlapped, 9H), 1.38 (s, 9H).

^13^C NMR (101 MHz, DMSO) δ (ppm): 170.93, 170.31, 169.78, 168.97, 155.88, 78.19, 61.69, 61.25, 55.26, 54.69, 51.94, 43.25, 41.87, 28.22. 

HRMS (ESI): m/z calculated for C_16_H_28_N_4_O_9_ [M + Na]^+^: 443.1754; found: 443.1754.

▪ Methyl Boc-l-phenylalanyl-l-serinyl-l-phenylalanyl-l-serinate (Compound **6b**)

We dissolved 0.352 g (1.0 mmol, 1 eq, obtained from benzyl ester) of Boc-l-Phe-l-Ser-OH, 0.333 g (1.1 mmol, 1.1 eq) of L-Phe-l-Ser-OMe×HCl, and 0.32 mL (2.3 mmol) of Et_3_N in ACN (20 mL). The reaction mixture was stirred and placed in the ice bath (−10 °C), and then 0.321 g (1.0 mmol, 1 eq) of TBTU was added in portions over a time span of 1 h. The ice bath was removed, and the reaction mixture was stirred at room temperature for 12 h. Then, the volatile components were removed under reduced pressure, and the reaction mixture was separated by means of flash column chromatography. The product was eluted using an increasing gradient of MeOH in chloroform from 0 to 25%. We obtained 0.334 g (0.56 mmol, 56% yield) of the product as a dense oil; Rf = 0.46 (10% MeOH in chloroform).

^1^H NMR (400 MHz, DMSO) δ (ppm): 8.43 (d, *J* = 7.6 Hz, 1H), 8.01 (d, *J* = 8.3 Hz, 1H), 7.94 (d, *J* = 7.7 Hz, 1H), 7.31–7.11 (m, 10H), 6.94 (d, *J* = 8.7 Hz, 1H), 5.10 (t, *J* = 5.7 Hz, 1H), 4.98 (t, *J* = 5.5 Hz, 1H), 4.68–4.60 (m, 1H), 4.39–4.33 (m, 1H), 4.32–4.25 (m, 1H), 4.23–4.15 (m, 1H), 3.74–3.59 and 3.63 (m and s overlapped, 5H), 3.58–3.42 (m, 2H), 3.08 (dd, *J* = 13.9, 4.3 Hz, 1H), 2.94 (dd, *J* = 13.8, 3.5 Hz, 1H), 2.81 (dd, *J* = 13.9, 9.1 Hz, 1H), 2.66 (dd, *J* = 13.8, 10.9 Hz, 1H), 1.27 (s, 9H).

^13^C NMR (101 MHz, DMSO) δ (ppm): 171.81, 171.15, 170.90, 169.79, 155.30, 138.33, 137.60, 129.30, 129.29, 128.04, 128.01, 126.27, 126.16, 78.10, 61.84, 61.19, 55.64, 54.89, 54.78, 53.56, 51.97, 37.42, 37.38, 28.16.

HRMS (ESI): m/z calculated for C_30_H_40_N_4_O_9_ [M + Na]^+^: 623.2693; found: 623.2687.

#### 4.2.4. Isomers of Methyl Boc-glycyl-dehydrophenyalanyl-glycyl-dehydrophenyalanine (Compounds **10**–**13**)

We added 0.152 mL (1.1 mM, 2.2 eq) of Et_3_N to the suspension of 0.174 g (0.5 mM, 1 eq) of Gly-^E^ΔPhe-OMe or Gly-^Z^ΔPhe-OMe trifluoroacetate in 4.0 mL of DCM, and then we stirred the mixture for 1.5 min. Then, 0.177 g (0.55 mM, 1.1 eq) of Boc-Gly-^E^ΔPhe-F was added, and the reaction was carried out for 4 h at room temperature. After the evaporation of the volatile components, the oily mixture was dissolved in EtOAc (25 mL) and washed with 2M HCl (3 × 2 mL), saturated KHCO_3_ (3 × 2 mL), and brine (1 × 3 mL). The organic layer was dried over anhydrous MgSO_4_, and the solvent was evaporated. Isomers with a purity exceeding 99.5% (as determined by HPLC) were separated by means of column chromatography on silica 60H (Merck, Darmstadt, Germany) using a gradient of ethyl acetate in benzene (from 5 to 65%).

- Acylation of Gly-^E^ΔPhe-OMe

▪ Methyl Boc-glycyl-(E)-dehydrophenyalanyl-glycyl-(E)-dehydrophenyalanine (Compound **10**)

We obtained 0.056 g (0.104 mmol, yield 21%) of tetrapeptide **10** after crystallization from the ethyl acetate-methanol(2:1)/hexane mixture. Mp = 205.5–207 °C (with decomposition).

^1^H NMR (400 MHz, DMSO) δ (ppm): 10.23 (s, 1H), 9.80 (s, 1H), 8.93 (broad t, *J* = 5.2 Hz, 1H), 7.51–7.17 (m, 10H), 7.15–7.07 (broad t, *J* = 5.4 Hz, 1H), 6.76 (s, 1H), 6.46 (s, 1H), 3.74 (broad s, 4H), 3.61 (s, 3H), 1.18 (s, 9H).

^13^C NMR (101 MHz, DMSO) δ (ppm): 168.98, 167.39, 165.29, 155.89, 134.27, 134.11, 131.72, 128.75, 128.41, 128.32, 128.06, 127.94, 127.42, 127.30, 118.47, 117.09, 77.96, 51.87, 43.02, 42.50, 27.94.

HRMS (ESI): m/z calculated for C_28_H_32_N_4_O_7_ [M + Na]^+^: 559.2169; found: 559.2156.

▪ Methyl Boc-glycyl-(Z)-dehydrophenyalanyl-glycyl-(E)-dehydrophenyalanine (Compound **11**)

We obtained 0.142 g (0.264 mmol, yield 53%) of tetrapeptide **11** after crystallization from the benzene-ethyl acetate(2:1)/hexane mixture. Mp = 168–170 °C (with decomposition).

^1^H NMR (400 MHz, DMSO) δ (ppm): 9.94 (s, 1H), 9.69 (s, 1H), 8.48 (t, *J* = 6.0 Hz, 1H), 7.63 (m, 2H), 7.45–7.16 (m, 9H), 7.10 (s, 1H), 6.68 (s, 1H), 3.87 (d, *J* = 6.0 Hz, 2H), 3.81 (d, *J* = 5.8 Hz, 2H), 3.61 (s, 3H), 1.34 (s, 9H).

^13^C NMR (101 MHz, DMSO) δ (ppm): 170.97, 167.69, 165.20, 165.07, 156.19, 134.10, 133.69, 129.85, 128.92, 128.80, 128.65, 128.56, 128.41, 127.95, 127.86, 127.58, 119.15, 78.29, 51.92, 43.62, 42.52, 28.16. 

HRMS (ESI): m/z calculated for C_28_H_32_N_4_O_7_ [M + Na]^+^: 559.2169; found: 559.2156.

- Acylation of Gly-^Z^ΔPhe-OMe

▪ Methyl Boc-glycyl-(E)-dehydrophenyalanyl-glycyl-(Z)-dehydrophenyalanine (Compound **12**)

We obtained 0.063 g (0.117 mmol, 24% yield) of tetrapeptide **12** after crystallization from the benzene-ethyl acetate(2:1)/hexane mixture. Mp = 194–196 °C.

^1^H NMR (400 MHz, DMSO) δ (ppm): δ 9.83 (s, 1H), 9.46 (s, 1H), 8.76 (t, *J* = 5.7 Hz, 1H), 7.74–7.63 (m, 2H), 7.44–7.14 (m overlapped with benzene protons, 9H), 6.77 (t, *J* = 5.9 Hz, 1H), 6.65 (s, 1H), 3.82 (d, *J* = 5.7 Hz, 2H), 3.71 (s, 3H), 3.57 (d, *J* = 5.9 Hz, 2H), 1.35 (s, 9H).

^13^C NMR (101 MHz, DMSO) δ (ppm): 168.70, 168.47, 165.31, 165.18, 155.73, 134.47, 133.11, 132.57, 131.67, 130.30, 129.55, 128.57, 128.25, 128.22, 127.04, 125.47, 117.46, 78.11, 52.27, 43.34, 42.61, 28.18.

HRMS (ESI): m/z calculated for C_28_H_32_N_4_O_7_ [M + Na]^+^: 559.2169; found: 559.2156.

▪ Methyl Boc-glycyl-(Z)-dehydrophenyalanyl-glycyl-(Z)-dehydrophenyalanine (Compound **13**)

We obtained 0.126 g (0.235 mmol, yield 47%) of tetrapeptide 13 after crystallization from the ethyl acetate-methanol(2:1)/hexane mixture. Mp = 145.5–147 °C.

^1^H NMR (400 MHz, DMSO) δ (ppm): 9.65 (s, 1H), 9.49 (s, 1H), 8.44 (t, *J* = 5.6 Hz, 1H), 7.70 (d, J = 6.8 Hz, 2H), 7.61 (d, J = 7.1 Hz, 2H), 7.37 (m, 6H), 7.26 (s, 1H), 7.24 (s, 1H), 7.06 (t, *J* = 5.6 Hz, 1H), 3.91 (d, *J* = 5.6 Hz, 2H), 3.74 (d overlapped, *J* = 5.6 Hz, 2H), 3.72 (s overlapped, 3H), 1.39 (s, 9H).

^13^C NMR (101 MHz, DMSO) δ (ppm): 170.06, 169.06, 165.37, 165.16, 156.14, 133.83, 133.16, 131.99, 130.29, 129.85, 129.55, 129.00, 128.88, 128.64, 128.59, 125.71, 78.34, 52.28, 43.79, 42.86, 28.24. The signal derived from one carbon atom in the aromatic/vinyl region of spectrum is not visible due to signals overlapping.

HRMS (ESI): m/z calculated for C_28_H_32_N_4_O_7_ [M + Na]^+^: 559.2169; found: 559.2156.

### 4.3. Crystallography

Relevant crystallographic data for the molecules and all geometrical information are summarized in Tables S2–S4 of the Supplementary Materials.

Single-crystal X-ray diffraction experiments were performed at 293 K on a KM4CCD diffractometer for peptides **3** and **15** and on an Xcalibur diffractometer (Rigaku Oxford Diffraction, Sevenoaks, Kent, UK) for peptide **14,** equipped with a CCD detector and a graphite monochromator (Rigaku Oxford Diffraction) with MoKα radiation. The reciprocal space was explored with ω scans. The reflections were measured with a radiation exposure time from 10 to 45 s, depending on diffraction intensities. The diffraction data were processed using CrysAlis CCD [60,61,62]. Structures for presented compounds **3**, **14**, and **15** were obtained in the triclinic crystal system and P¯1 for **15** and the orthorhombic crystal system, Fdd2 for **3** and the monoclinic crystal system, and P2_1_ for **14** and the space group (see Table S2 in the Supplementary Materials) with direct methods and refined with a full-matrix least squares method using the SHELXL14 program [63,64]. Lorentz and polarization corrections were applied. Non-hydrogen atoms were anisotropically refined. In structures, H atoms were refined using a riding model. The structure drawings were prepared using the Mercury program [65]. The crystallographic data for all compounds were deposited at the Cambridge Crystallographic Data Centre under supplementary publication numbers CCDC 2051490 for **15**, CCDC 2051491 for **3,** and CCDC 2051492 for **14**. These data can be obtained free of charge via www.ccdc.cam.ac.uk/structures/ (access date 3 December 2021) or from the Cambridge Crystallographic Data Centre, 12 Union Road, Cambridge CB2 1EZ, UK; fax: +44-1223-336-033; e-mail: deposit@ccdc.cam.ac.uk. 

## Figures and Tables

**Table 1:** The comparison of the synthetic methods for the preparation of desired tetrapeptides.

Compound	Synthetic Method	Isolated Yield	
**1a**	Dehydration of serine residues (MsCl, Et_3_N, Et_3_N × HCl, DBU in DMF at −10 °C, 2.5 h)	70% ^1^	
**1b**		73%	
**1c**	Coupling of dipeptide building blocks—mixed anhydride method (Isobutyl chloroformate, NMM in ACN or THF at −15 °C, overnight)	48%	
**3**		55%	
**4**	Coupling of dipeptide building blocks—guanidinium salt method (TBTU, Et_3_N in ACN at −10 °C, 12 h)	66%	
**6a**		59% ^2^	
**6b**		56% ^2^	
**10**	Acylation of dipeptide building block by acyl fluoride (1. TFFH, DIPEA in DCM at 8–9 °C, 0.5 h; 2. Boc-AA-ΔAA-F, Et_3_N in DCM at rt, 4 h)	21%	Total 74% ^3^
**11**		53%	
**12**		24%	Total 71% ^3^
**13**		47%	

^1^ The reaction performed without presence of equivalent of Et_3_N × HCl gave the product with the yield of 57%; ^2^ the reactions were performed without protection of hydroxyl group of serine; ^3^ the coupling of dipeptide building blocks using other coupling gents gave the products with low total yields: DCC—25–28%, TBTU—33–36%, isobutyl chloroformate—33–38%.

## Data Availability

The data of this study are presented within the study.

## Supplementary Materials

The following supporting information can be downloaded at: https://www.mdpi.com/article/10.3390/molecules27092611/s1, Table S1: The comparison of the synthetic pathways leading to the preparation of desired dehydropeptides, Table S2: Experimental details for compounds **3**, **14** and **15** (Crystallographic data), Table S3: Selected geometric parameters (Å, º) for **3**, **14** and **15**, Table S4: Selected hydrogen-bond parameters for **14** and **15**, Figure S1: 1H NMR spectra of the dipeptie Boc-l-Phe-l-Ser-OH: spectrum 1—pure diastereomer (obtained via hydrogenolysis of benzyl ester); spectrum 2—mixture of diastereomers (obtained via alkaline hydrolysis of methyl ester), Figure S2: 1H NMR spectra of the tetrapeptides **6a** (left) and **6b** (right): spectra 1—pure diastereomer; spectra 2—mixture of diastereomers. The amide and hydroxyl region of the spectrum is magnified, Figure S3: 1H NMR spectrum of compound **7** (coupling reaction of Boc-l-Phe-OH with L-Ser-OMe × HCl using T3P reagent), Figure S4: TLC chromatogram obtained from the dehydration reactions of Boc-Gly-l-Ser-Gly-l-Ser-OMe: line 1—substrate; line 2—reaction mixture with the addition of Et_3_N × HCl; line 3—reaction mixture without the addition of Et_3_N×HCl, Figure S5: TLC chromatograms of fractions obtained from column chromatography of crude product of dehydration of **6a**, Figure S6: HRMS (ESI) analysis of fraction 44 obtained from column chromatography of crude product of dehydration of **6a**, Figure S7: MS/MS analysis of peak characterized by mass of 503.1414 Da, Figure S8: MS/MS analysis of peak characterized by mass of 425.1641 Da, Figure S9: 1H NMR spectrum of one of Boc-l-Phe-Ser(OMes)-l-Phe-ΔAla-OMe—the side product of dehydration **6b** without presence of Et_3_N × HCl in the reaction mixture [46,47].

## Sample Availability

Samples of the synthesized compounds are available from the authors.

## References

[1] Nanda V., Koder R.L. (2010). Designing artificial enzymes by intuition and computation. Nat. Chem..

[2] Raffy Q., Ricoux R., Mahy J.-P., Ghang M., Ramel B. (2011). A new kind of eco-compatible hybrid biocatalyst for selective reactions: Artificial metalloenzymes. Focus on Catalysis Research: New Developments.

[3] Ward T.R. (2011). Artificial metalloenzymes based on the biotin−avidin technology: Enantioselective catalysis and beyond. Acc. Chem. Res..

[4] Raynal M., Ballester P., Vidal-Ferran A., van Leeuven P.W.N.M. (2014). Supramolecular catalysis. Part 2: Artificial enzyme mimics. Chem. Soc. Rev..

[5] Di Meo T., Ghattas W., Herrero C., Velours C., Minard P., Mahy J.-P., Ricoux R., Urvoas A. (2017). αRep A3: A versatile artificial scaffold for metalloenzyme design. Chem. Eur. J..

[6] Sanasiaume-Dagousset E., Urvoas A., Chelly K., Ghattas W., Maréchal J.-D., Mahy J.P., Ricoux R. (2014). Neocarzinostatin-based hybrid biocatalysts for oxidation reactions. Dalton Trans..

[7] Köhler V., Wilson Y.M., Lo C., Sardo A., Ward T.R. (2010). Protein-based hybrid catalysts—design and evolution. Curr. Opin. Biotechnol..

[8] Talbi B., Haquette P., Marel A., de Montigny F., Fosse C., Cordier S., Roisnel T., Jaouen G., Salamain M. (2010). (η^6^-Arene) ruthenium (II) complexes and metallo-papain hybrid as Lewis acid catalysts of Diels–Alder reaction in water. Dalton Trans..

[9] Bayat S., Tejo B.A., Abdulmalek E., Salleh A.B., Normi Y.M., Rahman M.B.A. (2014). Rational design of mimetic peptides aldo-ketoreductase enzyme as asymmetric organocatalysts in aldol reactions. RSC Adv..

[10] Yuan Z., Jin Z., Yingwu L. (2015). Rational design of hydrolases in protein scaffolds. Prog. Chem..

[11] Razkin J., Nilsson H., Baltzer L. (2007). Catalysis of the cleavage of uridine 3′-2,2,2-trichloroethylphosphate by a designed helix−loop−helix motif peptide. J. Am. Chem. Soc..

[12] McAllister K.A., Zou H., Cochran F.V., Bender G.M., Senes A., Fry H.C., Nanda V., Keenan P.A., Lear J.D., Saven J.G. (2008). Using α-helical coiled-coils to design nanostructured metalloporphyrin arrays. J. Am. Chem. Soc..

[13] Zastrow M.L., Peacock A.F.A., Stuckey J.A., Pecoraro V.L. (2011). Hydrolytic catalysis and structural stabilization in a designed metalloprotein. Nat. Chem..

[14] Armstrong C.T., Watkins D.W., Anderson J.L.R. (2013). Constructing manmade enzymes for oxygen activation. Dalton Trans..

[15] Drewniak M., Węglarz-Tomczak E., Ożga K., Rudzińska-Szostak E., Macegoniuk K., Tomczak J.M., Bejger M., Rypniewski W., Berlicki Ł. (2018). Helix-loop-helix peptide foldamers and their use in the construction of hydrolase mimetics. Bioorg. Chem..

[16] Ferrazzano L., Corbisiero D., Greco R., Potenza E., De Seriis G., Garelli A., Tolomelli A. (2019). Synthesis of α/β dipeptides containing linear or cyclic α-dehydro-β-amino acids as scaffolds for bioactive compounds. Amino Acids.

[17] Goodman C.M., Choi S., Shandler S., De Grado W.F. (2007). Foldamers as versatile frameworks for the design and evolution of function. Nat. Chem. Biol..

[18] Joaquin D., Lee M.A., Kastner D.W., Singh J., Morrill S.T., Damestedt G., Castle S.L. (2020). Impact of dehydroamino acids on the structure and stability of incipient 3_10_-helical peptides. J. Org. Chem..

[19] Jalan A., Kastner D.W., Webber K.G.I., Smith M.S., Price J.L., Castle S.L. (2017). Bulky dehydroamino acids enhance proteolytic stability and folding in β-hairpin peptides. Org. Lett..

[20] Shivhare A., Garg C., Priyam A., Gupta A., Sharma A.K., Kumar P. (2018). Enzyme sensitive smart inulin-dehydropeptide conjugate self assembles into nanostructures useful for targeted delivery of ornidazole. Int. J. Biol. Macromol..

[21] De Vries R.H., Viel J.H., Oudshoorn R., Kuipers O.P., Gerard Roelfes G. (2019). Selective modification of ribosomally synthesized and post-translationally modified peptides (RiPPs) through Diels–Alder cycloadditions on dehydroalanine sesidues. Eur. J. Chem..

[22] Broda M.A., Siodłak D., Rzeszotarska B. (2005). Conformational investigation of α,β-dehydropeptides. XV: N-acetyl-α,β-dehydroamino acid N ′N ′-dimethylamides: Conformational properties from infrared and theoretical studies. J. Pept. Sci..

[23] Latajka R., Jewgiński M., Makowski M., Krężel A. (2008). Conformational studies of hexapeptides containing two dehydroamino acid residues in positon 3 and 5 in peptide chain. J. Mol. Struct..

[24] Buczek A., Makowski M., Jewgiński M., Latajka R., Kupka T., Broda M.A. (2014). Toward engineering efficient peptidomimetics. screening conformational landscape of two modified dehydroaminoacids. Biopolymers.

[25] Wang T., Chao W., Li L., Zheng R., Sun D. (2020). Synthesis of dehydroamino acids and their applications in the drug research and development. Progress Chem..

[26] Siodłak D. (2015). α,β-Dehydroamino acids in naturally occurring peptides. Amino Acids.

[27] Levengood M.R., van der Donk W.A. (2006). Dehydroalanine-containing peptides: Preparation from phenylselenocysteine and utility in convergent ligation strategies. Nat. Prot..

[28] Cossec B., Cosnier F., Burgart M. (2008). Methyl mercapturate synthesis: An efficient, convenient and simple method. Molecules.

[29] Lenartowicz P., Makowski M., Oszywa B., Haremza K., Latajka R., Pawełczak M., Kafarski P. (2017). Addition of thiols to the double bond of dipeptide C -terminal dehydroalanine as a source of new inhibitors of cathepsin C. Biochimie.

[30] Lenartowicz P., Dziuk B., Zarychta B., Makowski M., Kafarski P. (2017). Michael additions to double bonds of esters of N-protected (s)-phenylalanyldehydroalanine (X-(s)-Phe-ΔAla-OMe) and its phosphonic acid counterpart (X-(s)-Phe-ΔAla-PO(OEt)_2_). Phosphorus Sulfur Silicon Relat. Elem..

[31] Lenartowicz P., Psurski M., Kotynia A., Pieniężna A., Cuprych M., Poniatowska K., Brasuń J., Kafarski P. (2021). Dipeptides of S-substituted dehydrocysteine as artzyme building blocks: Synthesis, complexing abilities and antiproliferative properties. Int. J. Mol. Sci..

[32] Bogart J.W., Bowers A.A. (2019). Dehydroamino acids: Chemical multi-tools for late-stage diversification. Org. Biomol. Chem..

[33] Wan Y., Zhu J., Wang W., Zhang Y. (2021). Synthesis of β-silyl α-amino Acids via visible-light-mediated hydrosilylation. Org. Lett..

[34] Ferreira P.M.T., Maia H.L.S., Monteiro L.S., Sacramento J. (2001). Michael addition of thiols, carbon nucleophiles and amines to dehydroamino acid and dehydropeptide derivatives. J. Chem. Soc. Perkin Trans. 1.

[35] Ferreira P.M.T., Maia H.L.S., Monteiro L.S. (2003). Synthesis of Non-Natural Amino Acids from N-(p-Tolylsulfonyl)-α,β-didehydroamino Acid Derivatives. Eur. J. Org. Chem..

[36] Kakkar S., Narasimham B. (2019). A comprehensive review on biological activities of oxazole derivatives. BMC Chem..

[37] Mhlongo J.T., Brasil E., de la Torre B.G., Albericio F. (2020). Naturally occurring oxazole-containing peptides. Mar. Drugs.

[38] Petersen J., Christensen K.E., Nielsen M.T., Mortenses K.T., Komnatnyy V.V., Nielsen T.E., Qvortrup K. (2018). Oxidative modification of tryptophan-containing peptides. ACS Comb. Sci..

[39] Zhu J., Mo J., Lin H.-Z., Chen Y., Sun H.-P. (2018). The recent progress of isoxazole in medicinal chemistry. Bioorg. Med. Chem. Lett..

[40] Siodłak D., Staś M., Broda M.A., Bujak M., Lis T. (2014). Conformational properties of oxazole-amino acids: Effect of the intramolecular N-H···N hydrogen bond. J. Phys. Chem. B.

[41] Staś M., Bujak M., Broda M.A., Siodłak D. (2016). Conformational preferences and synthesis of isomers Z and E of oxazole-dehydrophenylalanine. Biopolymers.

[42] Tsutsumi H., Kuroda T., Kimura H., Goto Y., Suga H. (2021). Posttranslational chemical installation of azoles into translated peptides. Nat. Commun..

[43] Ramesh R., Mishra R., Chandrasekaran S. (2010). An improved procedure for the synthesis of dehydroamino acids and dehydropeptides from the carbonate derivatives of serine and threonine using tetrabutylammonium fluoride. J. Pep. Sci..

[44] Tian X., Li L., Han J., Zhen X., Liu S. (2016). Stereoselectively synthesis and structural confirmation of dehydrodipeptides with dehydrobutyrine. SpringerPlus.

[45] Latajka R., Makowski M., Jewgiński M., Pawełczak M., Koroniak H., Kafarski P. (2006). Peptide *p*-nitrophenylanilides containing (*E*)-dehydrophenylalanine—synthesis, structural studies and evaluation of their activity towards cathepsin C. New. J. Chem..

[46] Makowski M., Lenartowicz P., Oszywa B., Jewgiński M., Pawełczak M., Kafarski P. (2015). Synthesis of dehydrodipeptide esters and their evaluation as inhibitors of cathepsin C. Med. Chem. Res..

[47] Shin C.G., Yonezawa Y., Takahashi M., Yoshimura J. (1981). Dehydropeptides II. The synthesis of dehydrodipeptides by direct coupling and base-catalyzed β-elimination. Bull. Soc. Chem. Jpn..

[48] Ferreira P.M.T., Maia G.L.S., Monteiro L.S., Sacramento J. (1999). High yielding synthesis of dehydroamino acid and dehydropeptide derivatives. J. Chem. Soc. Perkin Trans. 1.

[49] Ramesh R., De K., Chandrasekaran S. (2007). An efficient synthesis of dehydroamino acids and dehydropeptides from O-Cbz and O-Fmoc derivatives of serine and threonine. Tetrahedron.

[50] Makowski M., Rzeszotarska B., Kubica Z., Pietrzyński G. (1985). Synthesis of Peptides with α,β-Dehydroamino Acids, II. Synthesis of tert-butyloxycarbonyldipeptides of dehydroalanine and dehydrophenylalanine. Liebigs Ann. Chem..

[51] Makowski M., Rzeszotarska B., Kubica Z., Wieczorek P., Makowski M., Rzeszotarska B., Kubica Z., Wieczorek P. (1984). Synthesis of Peptides with α,β-Dehydroamino Acids, I Synthesis of *N*-benzyloxycarbonyl and *N*-trifluoroacetyl dipeptides of dehydroalanine and dehydrophenylalanine. Liebigs Ann. Chem..

[52] Murru S., Nefzi A. (2014). Combinatorial synthesis of oxazol-thiazole bis-heterocyclic compounds. ACS Comb. Sci..

[53] Phillips A.J., Uto Y., Wipf P., Reno M.J., Williams D.R. (2000). Synthesis of functionalized oxazolines and oxazoles with DAST and Deoxo-Fluor. Org. Lett..

[54] Kaiser D., Videnov G., Maichle-Mossmer C., Strahle J., Jung G. (2000). X-Ray structures and *ab initio* study of the conformational properties of novel oxazole and thiazole containing di- and tripeptide mimetics. J. Chem. Soc. Perkin Trans. 2.

[55] Latajka R., Jewgiński M., Makowski M., Krężel A. (2008). Conformational studies of hexapeptides containing two dehydroamino acid residues in positon 2 and 5 in peptide chain. Biopolymers.

[56] Gruß H., Feiner R.C., Mseya R., Schröder D.C., Jewgiński M., Müller K.M., Latajka R., Marion A., Seewald N. (2022). Peptide stapling by late-stage Suzuki–Miyaura cross-coupling. Beilstein J. Org. Chem..

[57] Jewgiński M., Latajka R., Krężel A., Haremza K., Makowski M., Kafarski P. (2013). Influence of solvents on conformation of dehydropeptides. J. Mol. Struct..

[58] Jewgiński M., Krzciuk-Gula J., Makowski M., Latajka R., Kafarski P. (2014). Conformation of dehydropentapeptides containing four achiral amino acid residues—controlling the role of L-valine. Beilstein J. Org. Chem..

[59] Jaremko M., Jaremko Ł., Mazur A., Makowski M., Lisowski M., Mazur A. (2013). Enhanced β-turn conformational stability of tripeptides containing ΔPhe in cis over trans configuration. Amino Acids.

[60] (2005). CrysAlis CCD, Version 1.171.33.57.

[61] (2005). CrysAlis RED, Version 1.171.33.57.

[62] Rigaku Oxford Difraction (2016). CrysAlisPro.

[63] Sheldrick G.M. (2008). A short history of SHELX. Acta Crystallogr. Sect. A.

[64] Sheldrick G.M. (2015). New features added to the refinement program SHELXL since 2008 are described and explained. Acta Crystallogr. Sect. C.

[65] Macrae C.F., Bruno I.J., Chisholm J.A., Edgington P.R., McCabe P., Pidcock E., Rodriguez-Monge L., Taylor R., van de Streek J., Wood P.A. (2008). New Features for the Visualization and Investigation of Crystal Structures. J. Appl. Crystallogr..

