# Supplementary material for: Synthesis of Tetrapeptides Containing Dehydroalanine, Dehydrophenylalanine and Oxazole as Building Blocks for Construction of Foldamers and Bioinspired Catalysts
Source: Molecules. 2022 Apr 19;27(9):2611. doi: 10.3390/molecules27092611 (PMC9102237; doi:10.3390/molecules27092611)
Supplement: Supplementary file 1 [file molecules-27-02611-s001.zip › molecules-1652145-supplementary.pdf]

Type of the Paper (Article)

Synthesis of tetrapeptides containing dehydroalanine, dehydrophenylalanine and oxazole as building blocks for construction of foldamers and bioinspired catalysts

Paweł Lenartowicz[1,*], Maarten Beelen[1,2], Maciej Makowski[1,†], Weronika Wanat[3], Błażej Dziuk[3] and Paweł Kafarski[4,*]

[1] Faculty of Chemistry, University of Opole, ul. Oleska 48, 45-052 Opole, Poland; † Maciej Makowski passed away
[2] Department of Chemistry, Odisee University of Applied Sciences, Gebroeders De Smetstraat 1, 9000 Ghent, Belgium; maarten.beelen@hotmail.com
[3] Faculty of Chemistry, Wrocław University of Science and Technology, Wybrzeże Wyspiańskiego 27, 50-370 Wrocław, Poland; weronika.wanat@pwr.edu.pl; blazej.dziuk@pwr.edu.pl
[4] Faculty of Agriculture and Forestry, Warmia and Mazury University of Olsztyn, pl. Łodzki 4, 10-957 Olsztyn; pawel.kafarski@uwm.edu.pl
* Correspondence: pawel.lenartowicz@uni.opole.pl pawel.kafarski@uwm.edu.pl

Supplementary Materials

1. Short description of mass spectrometry studies on products of dehydration reaction 4
2. Synthesis of substrates for the preparation of desired peptides 6
3. ^{1}H, ^{13}C NMR spectra 8
4. Crystallographic data 19

Table S1. The comparison of the synthetic pathways leading to the preparation of desired dehydropeptides.

Compound	Advantages	Problems
Tetrapeptides **1a** and **1b**	a) total dehydration of serine residues in the presence of MsCl, DBU, $Et_3N\times HCl$ provided dehydropeptides wih yield of 70-73%<br>b) incorporation of ΔAla residues is performed at the end of synthetic pathway, thus the risk of polymerization is reduced<br>c) activation of ΔAla residue in coupling reaction is omitted	a) dehydration reaction performed without presence of Et_3NxHCl provide the mixture partially dehydrated and activated peptides. Thus chromatographic separation of the reaction mixture is complicated
Tetrapeptides	a) the dehydropeptides **1c** and **3** were	a) the activation of ΔAla residue by variety of

**1c** and **3**	obtained through coupling of dipeptide building blocks using mixed anhydride methods with isobutyl chloroformate. This method seems to be most suitable for the activation of dehydroalanine residue and provide desired products with moderate yield of 48-55%	coupling agent is not effective and provides the products with low yield<br>b) the preparation of substrate containing ΔAla residue, eg. Boc deprotection with TFA, can be accompanied with polymerization
Dipeptides **5**	a) the application of TBTU coupling agent, together with preactivation of carboxyl group followed by addition of amine component provided desired products with yield up to 86%<br>b) the protection of serine hydroxyl group is not necessary for coupling reaction	a) the application of T3P coupling agent provided acylation of serine hydroxyl group (compound **7**). Thus, the protection of hydroxyl group is required for this particular coupling reaction
Carboxyl group deprotection of dipeptides **5**	a) hydrogenolysis of dipeptide benzyl ester in the presence of Pd/C catalyst provided carboxylic acid with quantitative yield and prevented dipeptide racemization	a) alkaline hydrolysis of dipeptide methyl ester was accompanied with peptide racemization
Tetraptides **6**	a) the application of TBTU coupling agent and slight excess of amine component (10-20%) provided tetrapeptide with satisfactory yield of 56-59%<br>b) the protection of serine hydroxyl group is not necessary for coupling reaction	a) preactivation should be avoided due to autocondensation of the substrate<br>b) the acylation of serine hydroxyl group can occur and reaction yield is reduced
Tetrapeptides **10-13**	a) the application of *E*-dehydrophenylalanyl fluoride in acylation reaction of E or Z dehydrophenylalanynyl-containing dipeptide provides the access to the all four geometrical isomers which are easily separated by means of flash column chromatography<br>b) the reaction does not give side products and products are obtained with total yield of 71-74%	a) the use of DCC, TBTU, and mixed carbonic-carboxylic anhydrides, gave low yields (25-28%, 33-36% and 33-38%, respectively) and impure products

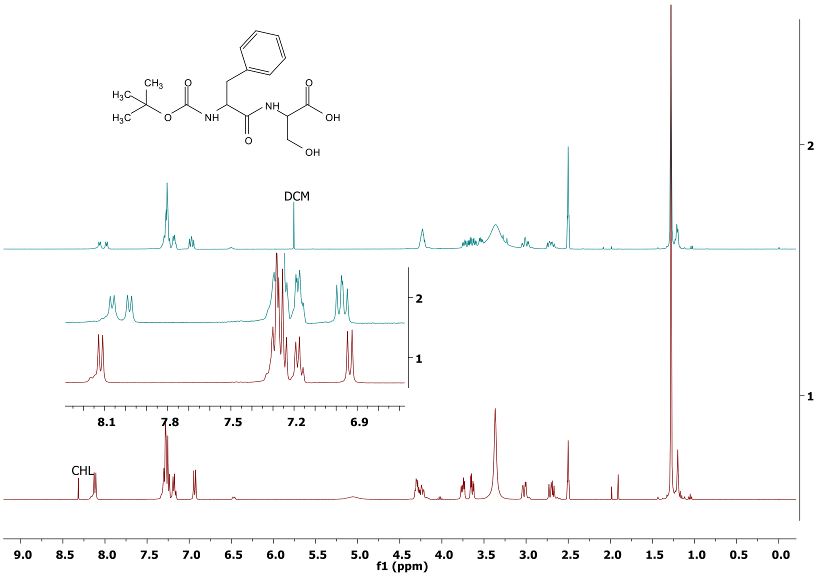

Figure S1. ^{1}H NMR spectra of the dipeptie Boc-L-Phe-L-Ser-OH: spectrum 1 – pure diastereomer (obtained via hydrogenolysis of benzyl ester); spectrum 2 –mixture of diastereomers (obtained via alkaline hydrolysis of methyl ester). The amide region of the spectrum is magnified.

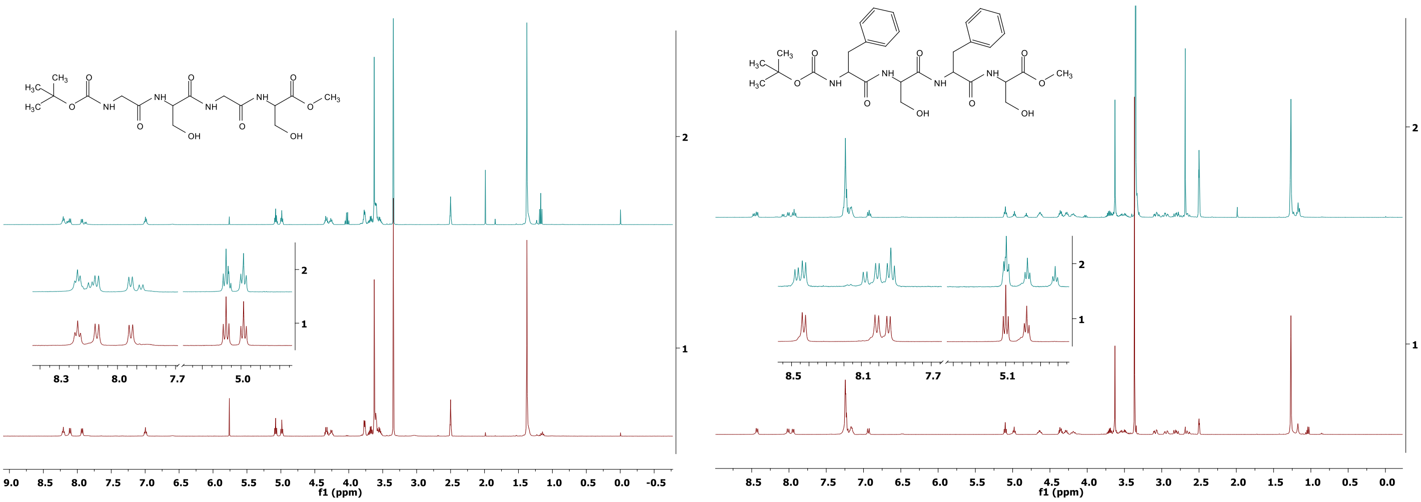

Figure S2. ^{1}H NMR spectra of the tetrapeptides **6a** (left) and **6b** (right): spectra 1 – pure diastereomer; spectra 2 –mixture of diastereomers. The amide and hydroxyl region of the spectrum is magnified.

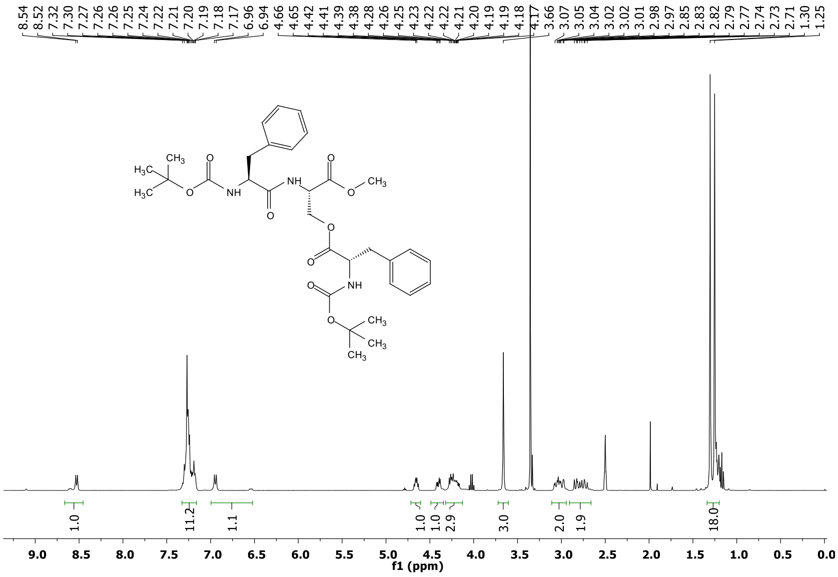

Figure S3. ^{1}H NMR spectrum of compound **7** (coupling reaction of Boc-L-Phe-OH with L-Ser-OMe×HCl using T3P reagent).

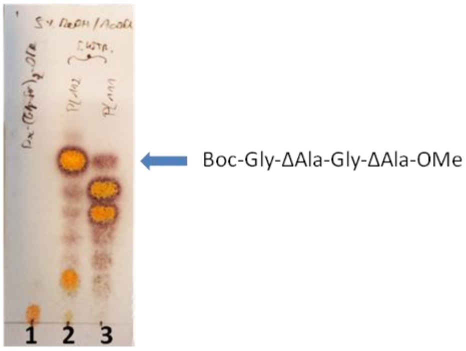

Figure S4. TLC chromatogram obtained from the dehydration reactions of Boc-Gly-L-Ser-Gly-L-Ser-OMe: line **1** – substrate; line **2** – reaction mixture with the addition of Et_3N×HCl; line **3** – reaction mixture without the addition of Et_3N×HCl.

1. Short description of mass spectrometry studies on products of dehydration reaction

The mixture obtained by dehydration of Boc-Gly-L-Ser-Gly-L-Ser-OMe (**6a**) without addition of triethylamine hydrochloride was separated by flash chromatography providing 22% (the worst result) of Boc-Gly-ΔAla-Gly-ΔAla-OMe (fractions 29-41, Figure S5).

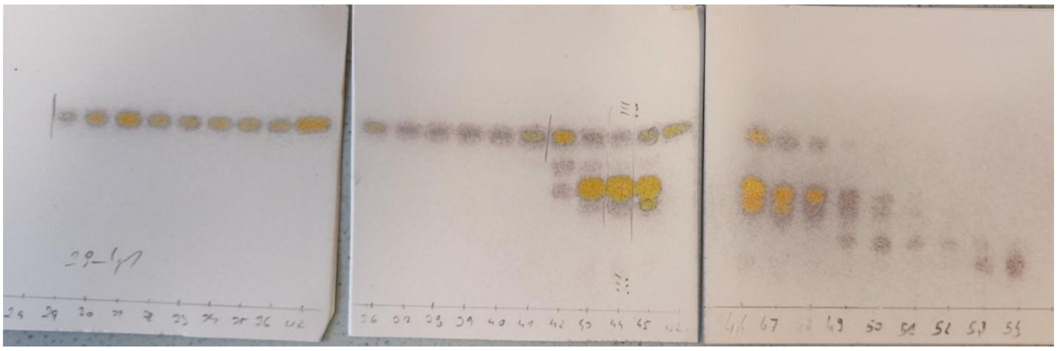

Figure S5. TLC chromatograms of fractions obtained from column chromatography of crude product of dehydration of **6a.**

The fraction 44 was analyzed by high resolution mass spectrometry with electrospray ionization (HRMS-ESI) to find out the possible side-products created during the dehydration. The three major peaks are observed on the MS spectrum with a mass to charge ratios (m/z) being: 503.1414, 425.1641 and 407.1549 Da (Figure S6). The structure determination based on the possible reaction products and fragmentation pattern of appropriate molecular ions (MS/MS analysis) reveals that peak 503.1414 Da comes from the sodium ion adduct of monomesyalated product Boc-Gly-Ser(OMes)-Gly-ΔAla-OMe $(M+Na)^+$ (structure **S1**) which has a monoisotopic mass of 503.1418 Da. The analysis of the MS/MS spectrum of that peak confirms this, since product is expected to fragmentate into structures **S2** (mass 407.1548 Da), **S3** (mass 351.0917 Da) and **S4** (mass 307.1031 Da) which are visible on the MS/MS spectrum (Figure S7). The recorded peak characterized by mass of 425.16.41 Da and its fragmentation pattern (structures **S6** and **S7**) confirm that the second main product is Boc-Gly-Ser-ΔAla-OMe (structure **S5**; Figure S8). In a similar manner structure of desired reaction product (compound **1a)** was determined. The fragmentation pattern for peak characterized by mass of 407.1549 Da (structure **S2**) is the same as for structure **S1**.

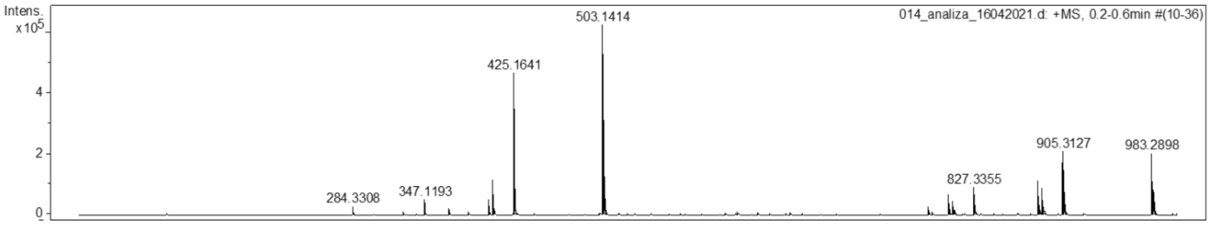

Figure S6. HRMS (ESI) analysis of fraction 44 obtained from column chromatography of crude product of dehydration of **6a**

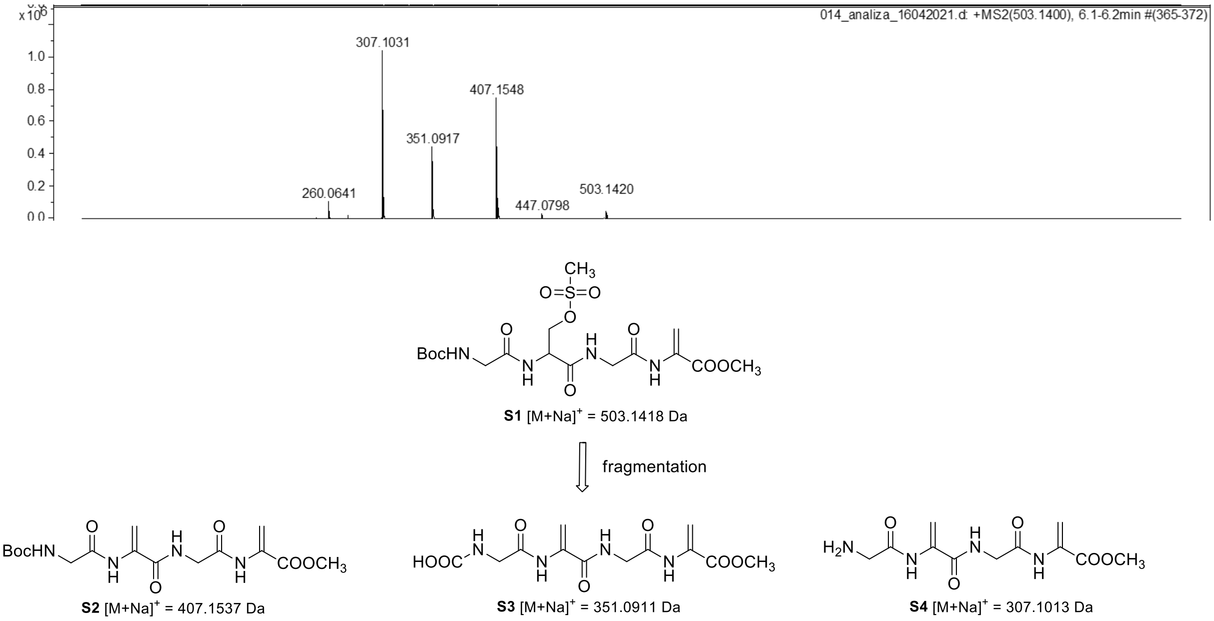

Figure S7. MS/MS analysis of peak characterized by mass of 503.1414 Da.

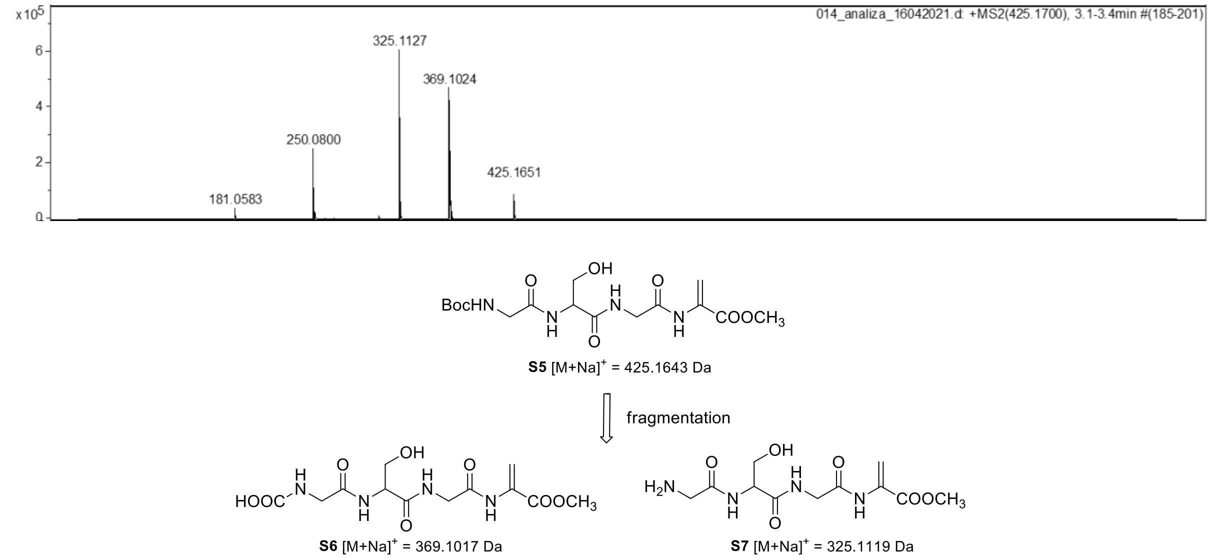

Figure S8. MS/MS analysis of peak characterized by mass of 425.1641 Da.

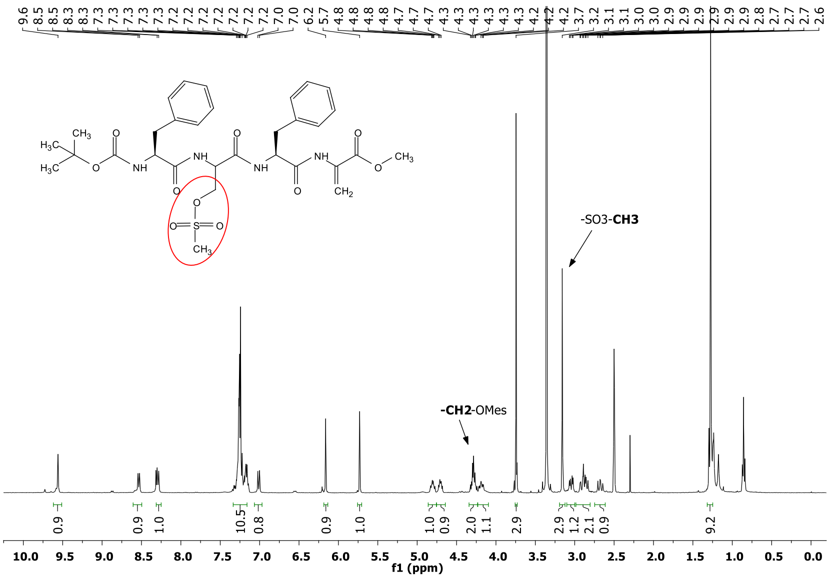

Figure S9. ^{1}H NMR spectrum of one of Boc-L-Phe-Ser(OMes)-L-Phe-ΔAla-OMe – the side product of dehydration **6b** without presence of Et_3N×HCl in the reaction mixture.

2. Synthesis of substrates for the preparation of desired peptides

Synthesis of oxazole building block (compound ***14****)* [46,47]

Boc-Gly-Ozl-COOMe: Boc-Gly-Ser-OMe (1.75 g; 6.3 mmol; 1 eq,) was dissolved in dry dichoromethane (10 ml) under argon atmosphere and cooled to -70°C. Then the solution of DAST (1 mL; 7.6 mmol 1.2 eq,) in dichoromethane (3 ml) was added dropwise. The reaction was stirred for 1 h and DBU (3.4 mL; 22.7 mmol; 3.6 eq) was added dropwise at -40°C. The reaction mixture change color to orange and mixing was continued for 1 h. $CBrCl_3$ (2.24 mL, 22.7 mmol; 3.6 eq) was added dropwise at -20°C, the formation of brown liquid was observed. The reaction mixture was stirred overnight at room temperature then saturated solution of $NaHCO_3$ (10 ml) was added. The product was extracted with ethyl acetate, dried over Na_2SO_4, filtered and concentrated. The resulting brown oil was purified by column chromatography (EtOAc:$CHCl_3$, 3:2, v/v), Rf = 0.25. The fractions containing product were evaporated and the yellow oil start crystallize. The recrystallization of the product from diethyl ether/hexane mixture at 4°C gave pale yellow crystals which were analyzed by NMR and X-Ray techniques.

Boc-glycyl-(E)-dehydrophenylalnine fluoride

Boc-Gly-^{E}ΔPhe-F: To the cold (7°C) solution of Boc-Gly-^{E}ΔPhe (1.6 g; 5 mM, 1.0 eq) and diisopropylethylamine (1.71 mL; 5 mM 1.0 eq) in dichloromethane (20 mL) tetramethylfluoroformamidynium hexaflouorophosphate (TFFH; 1.452 g; 5.5 mM, 1.1 eq) was added. The mixture was stirred at the temperature range of 8–9°C for 30-35 min. Then DCM was evaporated to the volume of 4 mL and diethyl ether (250 mL) was added. Resulting precipitate was collected by filtration. The second portion of the product was obtained by evaporation of crystallization mixture to the volume of 30 mL and addition of hexane. The crystallization was performed at 5°C. In that manner 1.34 g (83% of yield) of the product was obtained. It was used directly in further synthesis. M.p. = 127–128.5°C Elemental analysis calculated for $C_{16}H_{19}N_2O_4F$ C 59.62; H 5.94 and N 8.69%; found: C 59.85; H 5.79 and N 8.91%.

Boc-Gly-^{E}ΔPhe-OMe and **Boc-Gly-^{Z}ΔPhe-OMe**: The solution of Boc-Gly-^{E}ΔPhe or Boc-Gly-^{Z}ΔPhe (1.60 g; 5 mmol, 1.0 eq) and DMTMM (1.66 g; 6.0 mmol 1.2 eq) in dry methanol (15 mL) was stirred for 20 min. at 30°C followed by addition of NMM (0.665 mL; 6 mmol, 1.2 eq). The obtained mixture was

stirred for additional 7 h at the same temperature then methanol was evaporated and the resulting oil dissolved in ethyl acetate (25 mL). The solution was washed successively with: 1 M hydrochloric acid (4x3 mL), saturated $KHCO_3$ (4x3 mL), and brine (1x3 mL). Organic layer was dried over anhydrous magnesium sulphate and the solvent evaporated. Isomers were separated with purity exceeding 99.5% (by HPLC) by means of flash column chromatography using gradient of ethyl acetate in benzene (from 5-20%).

Boc-Gly-$^{E/Z}$ΔPhe-OMe: The separated isomers were crystallized from ethyl acetate-methanol (10:1)/hexane mixture. 0.938 g (56% yield, m.p = 131-133°C) of isomer E and 0.624 g (37% yield, m.p = 120-122°C) of isomer Z were obtained.

Boc-Gly-^{Z}ΔPhe-OMe: The product was crystallized from ethyl acetate-benzene (2:1)/hexane mixture. 1.521g (91% yield, m.p = 119.5-122 °C) of isomer Z was obtained.

Boc-L-Ser-OBz: To the stirred solution of Boc-L-Ser-OH (3.078 g, 15.0 mmol, 1 eq) in MeOH (30 mL) Cs_2CO_3 (2.444 g, 7.5 mmol, 0.5 eq) was added. After 30 minutes the volatile components was removed under reduced pressure. The residue was dissolved in DMF (50 mL), and benzyl bromide (2.68 mL, 22.5 mmol, 1.5 eq) was quickly added (at the brining of the reaction the solution has gel consistency). The reaction was continued for 6 hours. The volatile components were removed under reduced pressure and the residue was dissolved in EtOAc (150 mL). The organic phase was washed with brine (3x5 mL), 1M HCl (3×5 mL), saturated solution of $KHCO_3$ (3×5 mL) and again with brine (3×5 mL). Then the organic fraction was dried over $MgSO_4$ and solvent was evaporated. The crude product containing benzyl bromide was purified by means of flash column chromatography. First fraction was eluted with the mixture of hexane in toluene (10% v/v) and then the elution was changed to the gradient mode using mixture of EtOAc in hexane (from 0 to 50% v/v). The product (3.230 g, 10.9 mmol, yield 73%) was crystallized from EtOAc/hexane mixture.

L-Ser-OBz*HCl: Boc-L-Ser-OBz (2.953 g, 10 mmol) was dissolved in MeOH (21 mL) and was palced in ice bath. Then concentrated hydrochloric acid (4.25 mL) was added dropwise. The ice bath was removed and the reaction mixture was stirred for 3 hours. Then the isopropanol (10 mL) was added and the volatile components were removed under reduced pressure at 55°C. The oily residue was coevaporated with isopropanol three times (3x10 mL). The product (1.592, 6.9 mmol, yield 69%) was crystallized from H_2O/iPr-OH/acetone mixture.
Boc-L-Phe-L-Ser-OBz: The solution of Boc-L-Phe-OH (0.796 g, 3.0 mmol, 1 eq) and Et_3N (0.46 mL, 3.3 mmol, 1.1 eq) in ACN (10 mL) was placed in ice bath (-10°C) and then TBTU (0.963 g, 3.0 mmol, 1 eq) was added. After 10 minutes of preactivation the solution obtained by dissolving of L-Ser-OBz*HCl (0.730 g, 3.15 mmol, 1.05 eq) and Et_3N (0.46 mL, 3.3 mmol, 1.05 eq) in ACN (10 mL) was added dropwise. Then the ice bath was removed and reaction was stirred overnight at room temperature. The volatile components were removed under reduced pressure and residue was purified by means of flash column chromatography. The product was eluted with increasing gradient of EtOAc in hexane from 10% to 100%. The product (1.141 g, 2.58 mmol, 86%) was obtained as colorless oil.

The other coupling reactions with TBTU which were performed within this study were done in an analogical manner.

Boc-L-Phe-L-Ser-OH: Boc-L-Phe-L-Ser-OBz (1.062 g, 2.4 mmol) was dissolved in EtOH (20 mL) under argon atmosphere and placed in ice bath. Then the catalyst Pd/C 10% (0.074 g, 0.072 mmol, 3% mol) was added and argon was replaced by hydrogen. The reaction mixture was stirred for 3 hours. Then flask was purged with nitrogen gas and reaction mixture was filtrate through syringe filter. The volatile component was removed under reduced pressure. The residue was dissolved in EtOAc (80

mL) and washed with 1M HCl (3x5 mL) and brine (3x5 mL). The evaporation of the solvent to the dryness gave white amorphous solid product (0.845 g, quantitative yield).

3. ^{1}H, ^{13}C NMR spectra

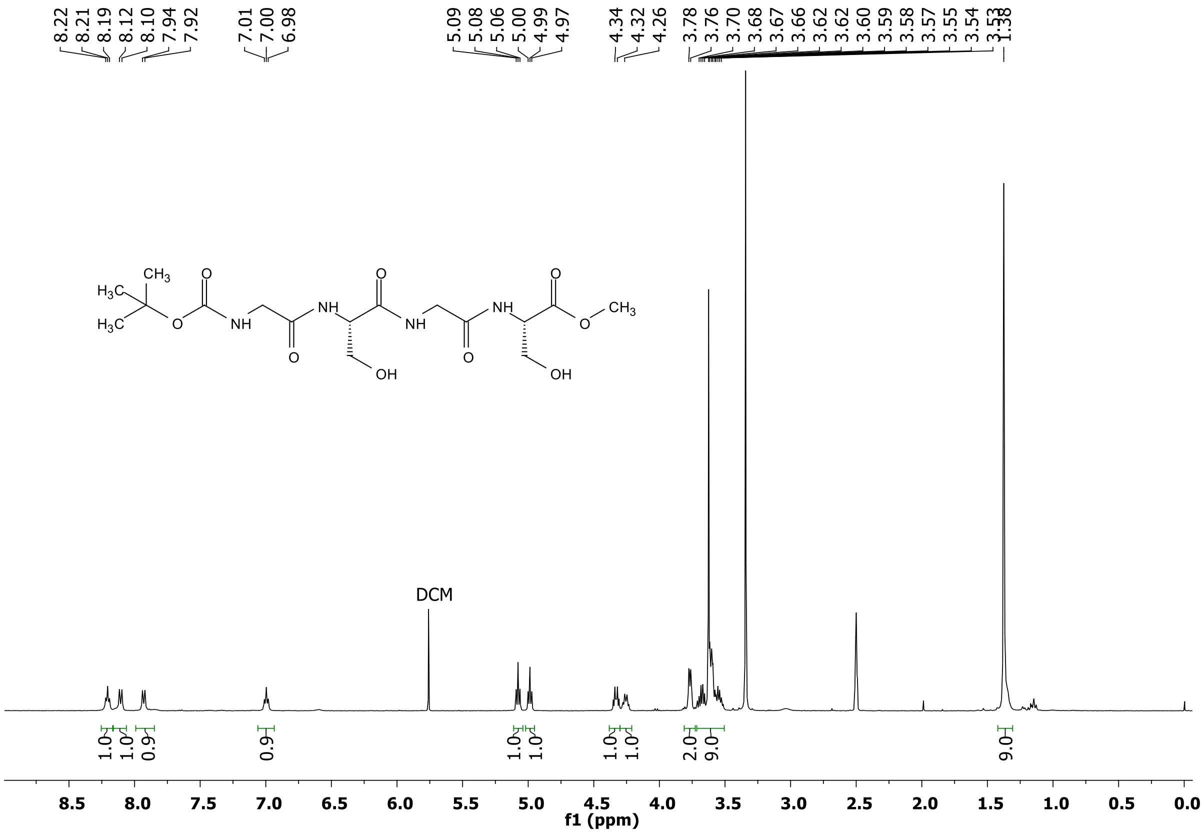

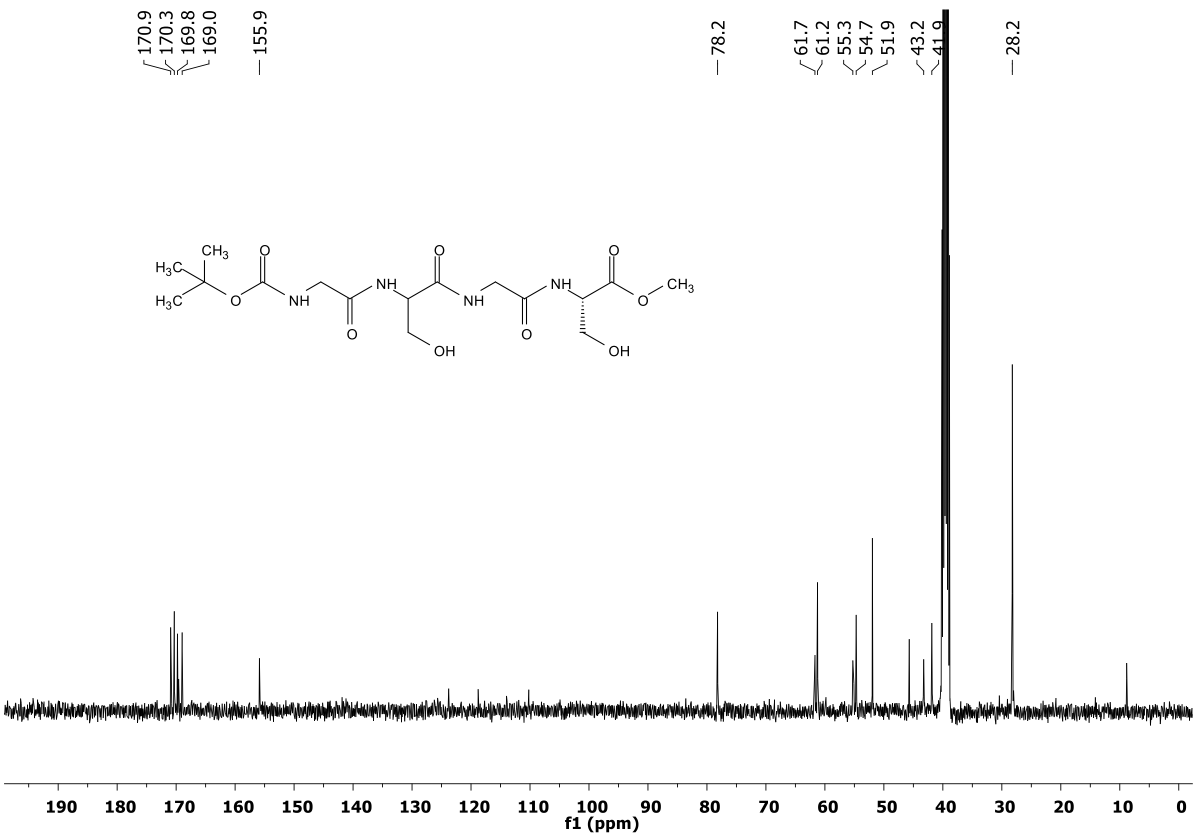
170.9
170.3
169.8
169.0
155.9
78.2
61.7
61.2
55.3
54.7
51.9
43.2
41.9
28.2
f1 (ppm)

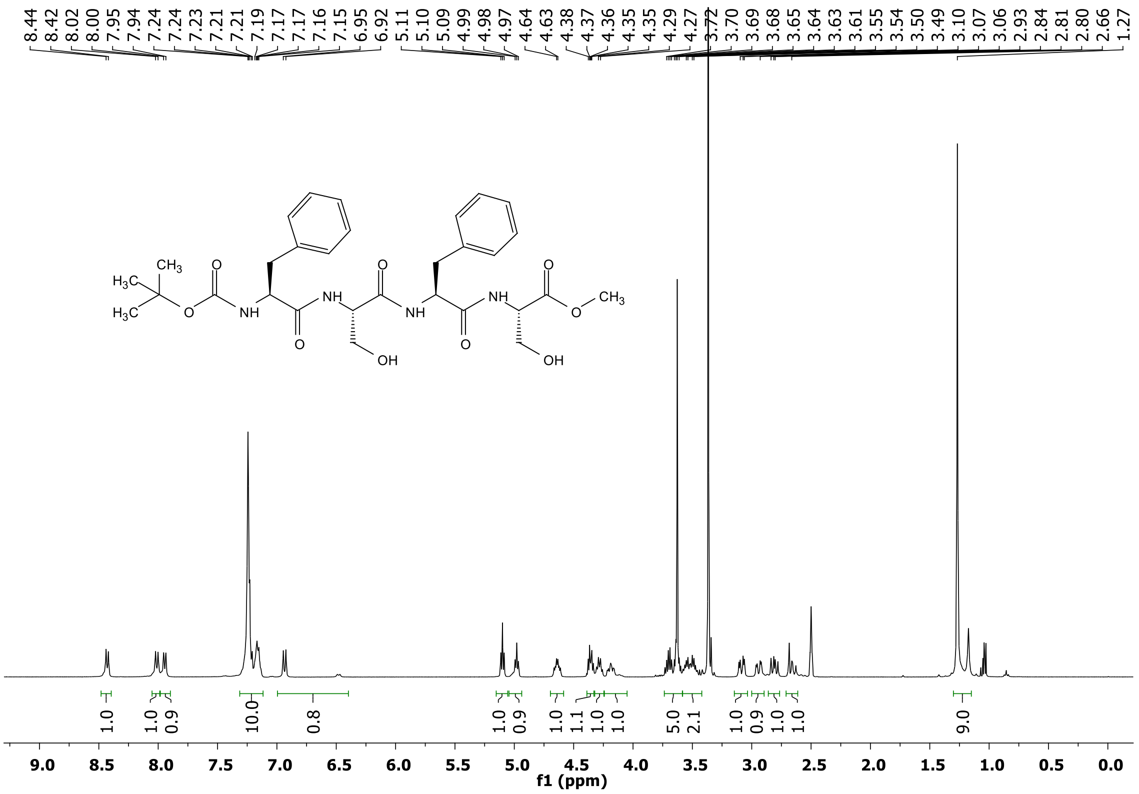
8.44
8.42
8.02
8.00
7.95
7.94
7.24
7.24
7.23
7.21
7.21
7.19
7.17
7.17
7.16
7.15
6.95
6.92
5.11
5.10
5.09
4.99
4.98
4.97
4.64
4.63
4.38
4.37
4.36
4.35
4.35
4.29
4.27
3.72
3.70
3.69
3.68
3.65
3.64
3.63
3.61
3.55
3.54
3.50
3.49
3.10
3.07
3.06
2.93
2.84
2.81
2.80
2.66
1.27
f1 (ppm)

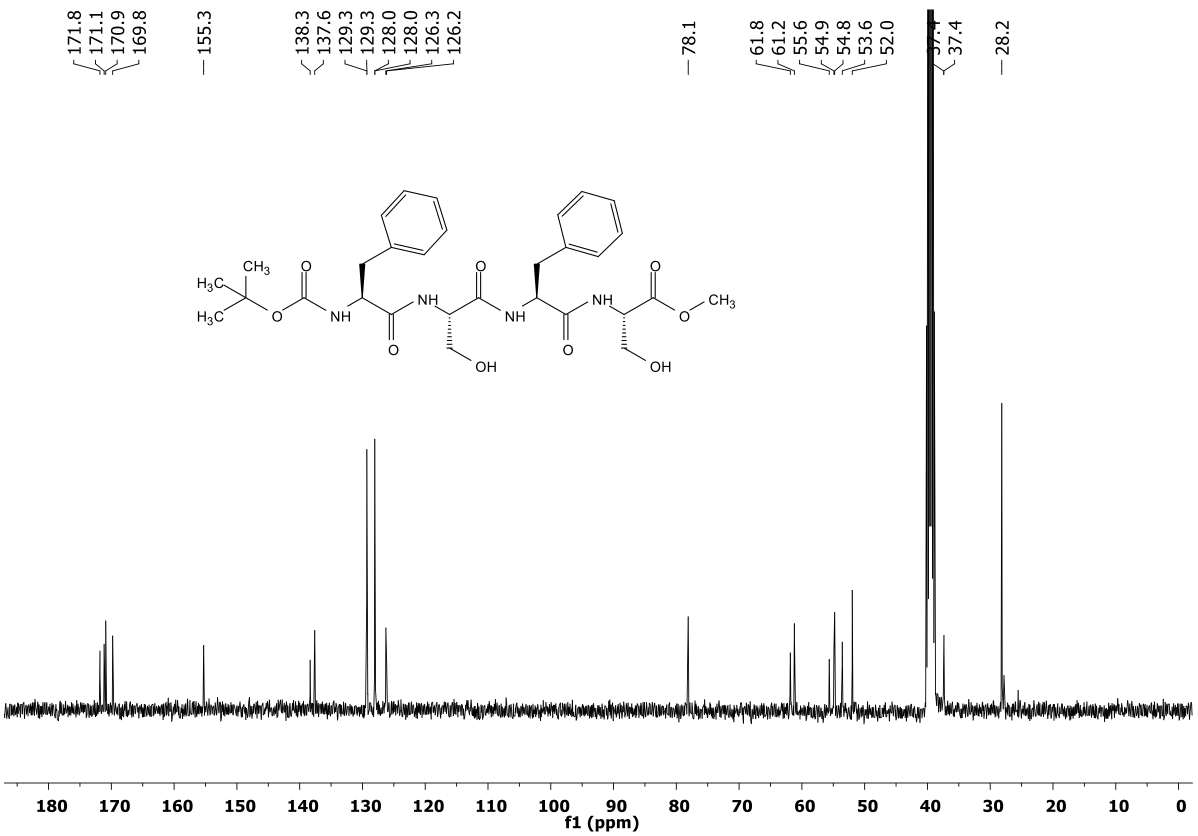
171.8
171.1
170.9
169.8
155.3
138.3
137.6
129.3
129.3
128.0
128.0
126.3
126.2
78.1
61.8
61.2
55.6
54.9
54.8
53.6
52.0
37.4
28.2
CH3
H3C
H3C
O
NH
OH
180 170 160 150 140 130 120 110 100 90 80 70 60 50 40 30 20 10 0
f1 (ppm)

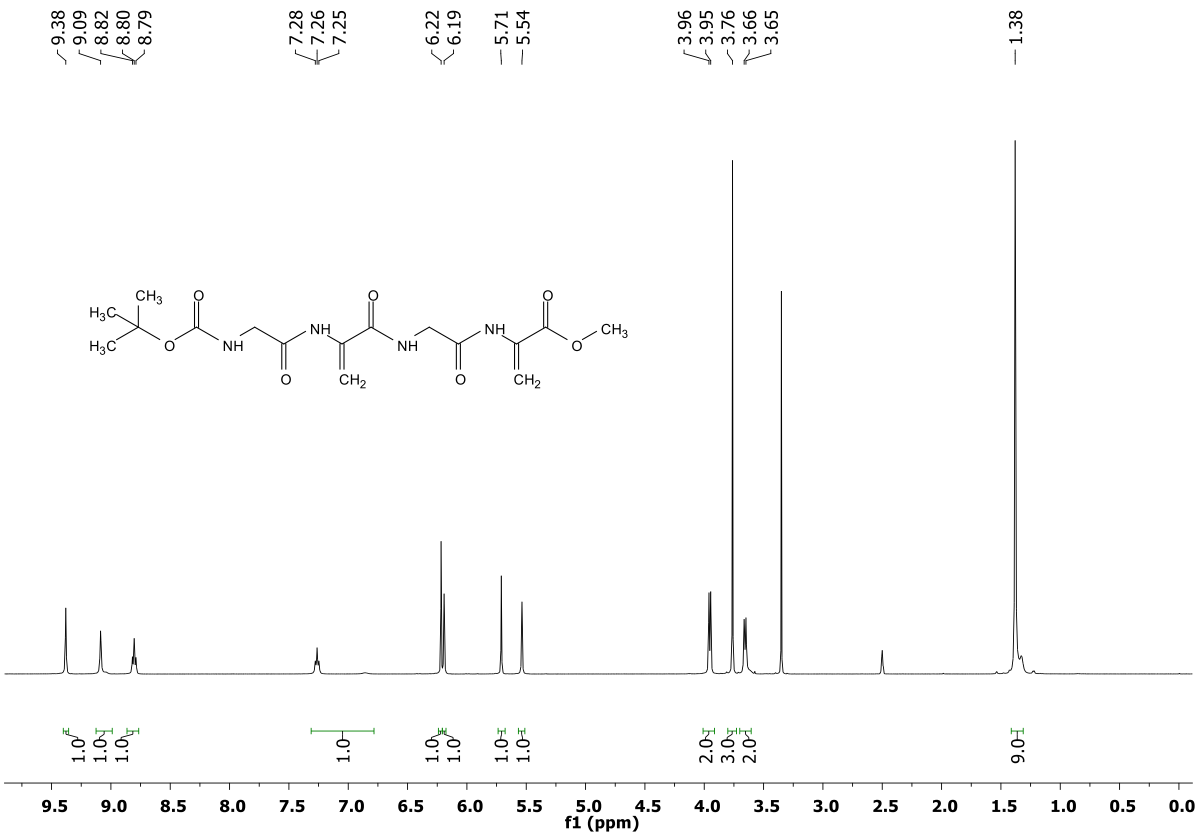
9.38
9.09
8.82
8.80
8.79
7.28
7.26
7.25
6.22
6.19
5.71
5.54
3.96
3.95
3.76
3.66
3.65
1.38
CH3
H3C
H3C
O
NH
CH2
1.0
1.0
1.0
1.0
1.0
1.0
1.0
1.0
2.0
3.0
2.0
9.0
9.5 9.0 8.5 8.0 7.5 7.0 6.5 6.0 5.5 5.0 4.5 4.0 3.5 3.0 2.5 2.0 1.5 1.0 0.5 0.0
f1 (ppm)

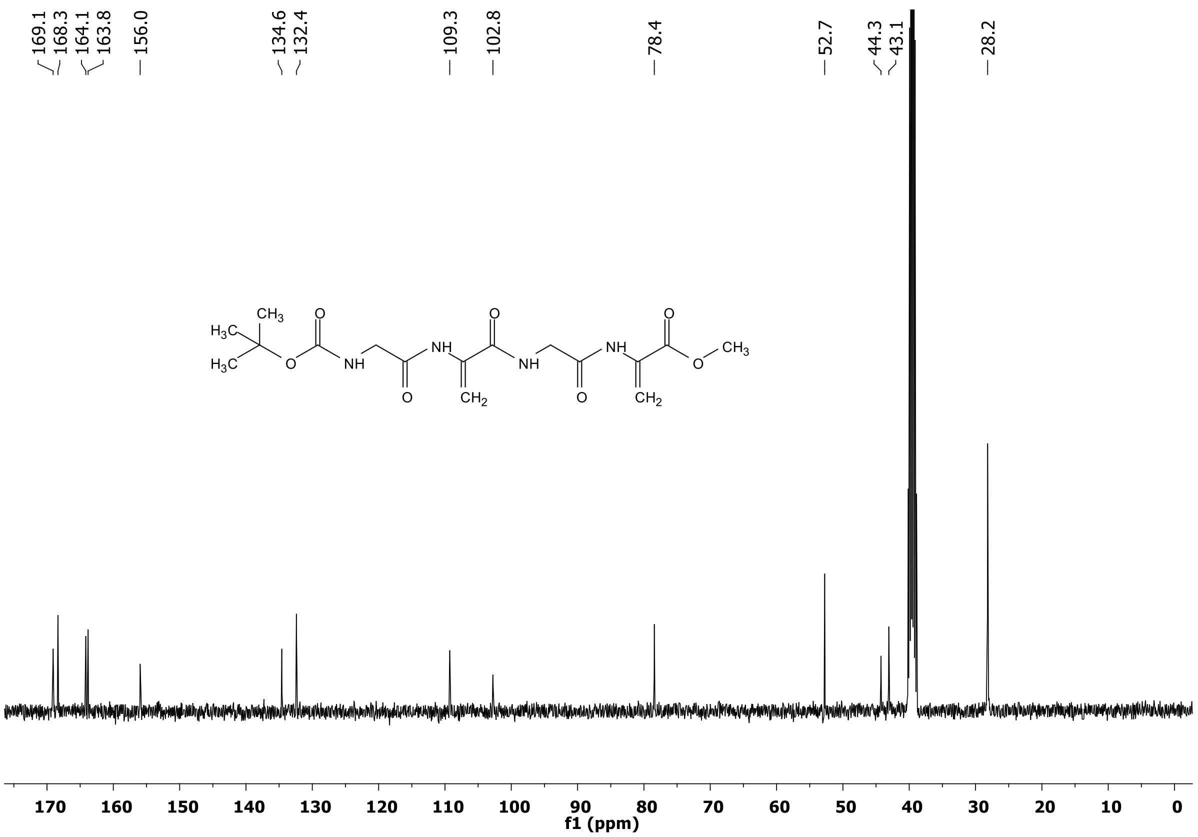
169.1
168.3
164.1
163.8
156.0
134.6
132.4
109.3
102.8
78.4
52.7
44.3
43.1
28.2
f1 (ppm)

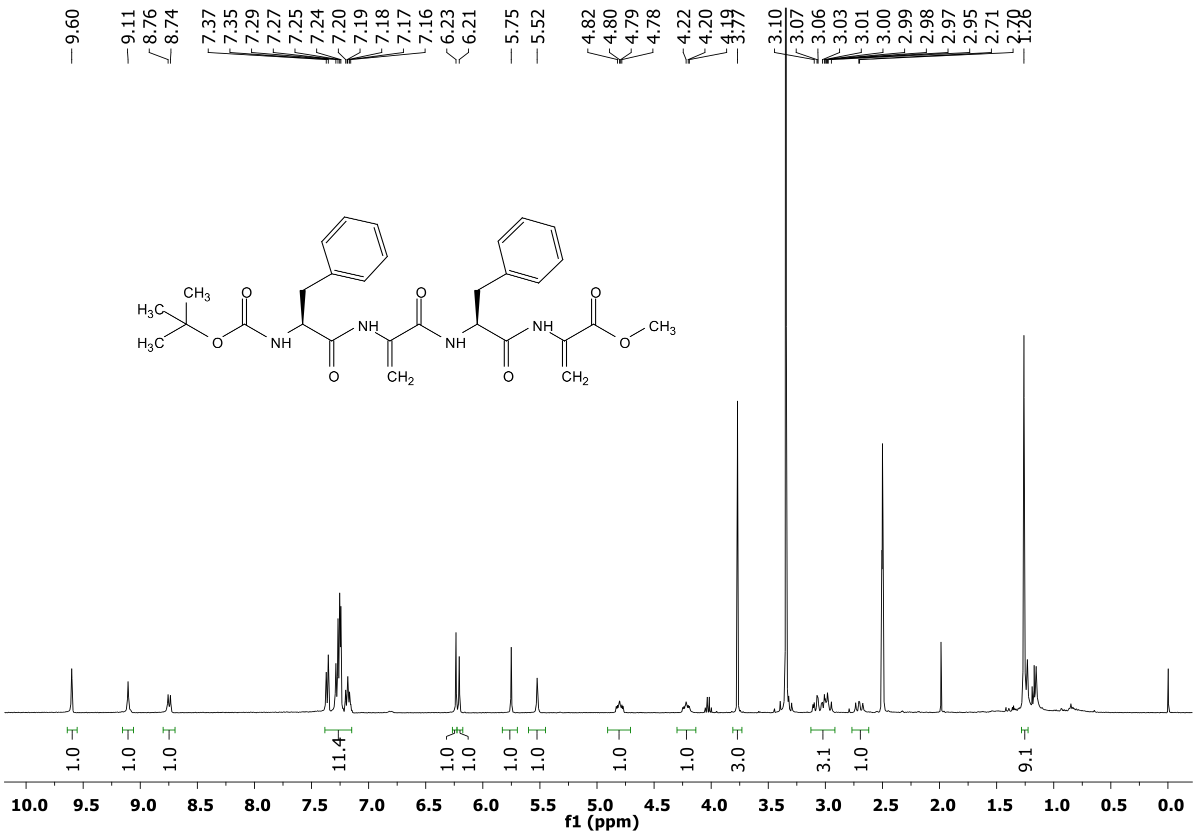
9.60
9.11
8.76
8.74
7.37
7.35
7.29
7.27
7.25
7.24
7.20
7.19
7.18
7.17
7.16
6.23
6.21
5.75
5.52
4.82
4.80
4.79
4.78
4.22
4.20
4.19
3.77
3.10
3.07
3.06
3.03
3.01
3.00
2.99
2.98
2.97
2.95
2.71
2.70
1.26
1.0
1.0
1.0
11.4
1.0
1.0
1.0
1.0
1.0
1.0
3.0
3.1
1.0
9.1
f1 (ppm)

Due to high level of a background noise, peaks derived from carbon atoms of ΔAla residue are poorly visible on the NMR spectrum of compound **1b**.

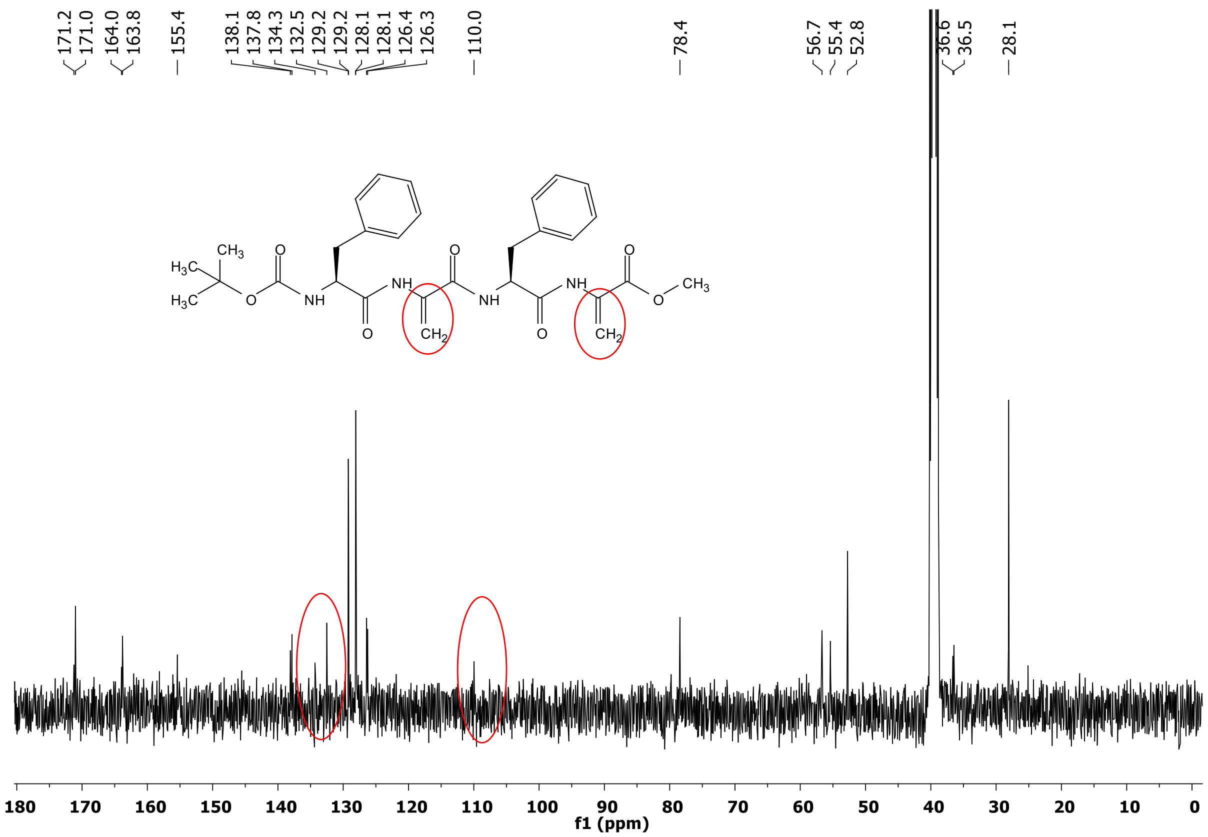

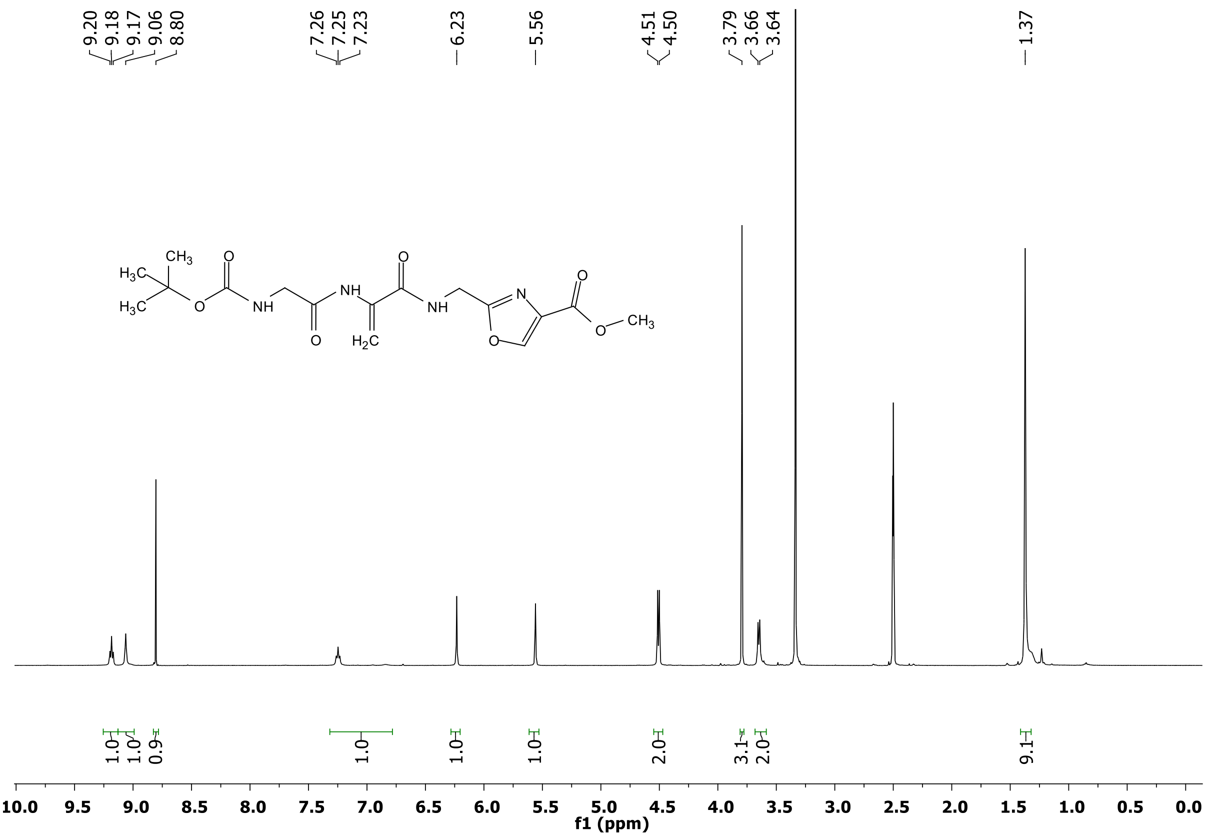

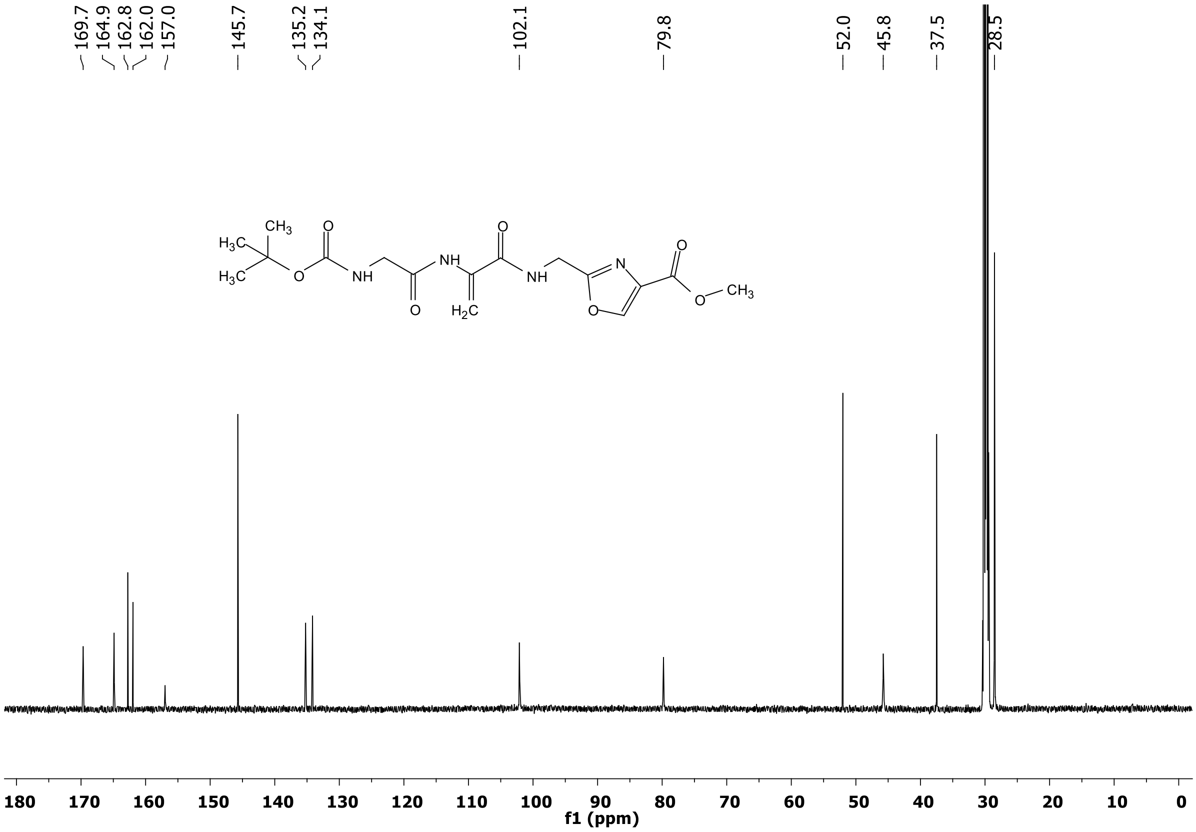
169.7
164.9
162.8
162.0
157.0
145.7
135.2
134.1
102.1
79.8
52.0
45.8
37.5
28.5
f1 (ppm)

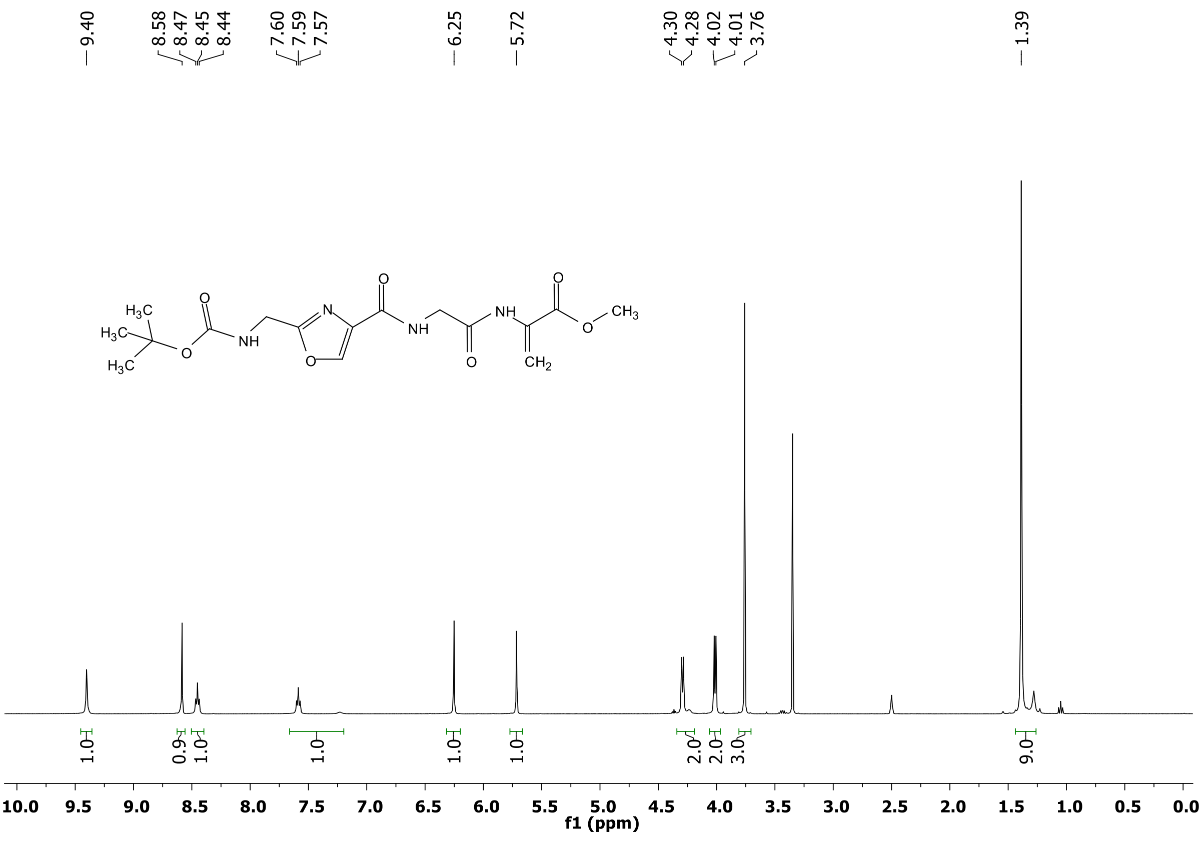
9.40
8.58
8.47
8.45
8.44
7.60
7.59
7.57
6.25
5.72
4.30
4.28
4.02
4.01
3.76
1.39
f1 (ppm)

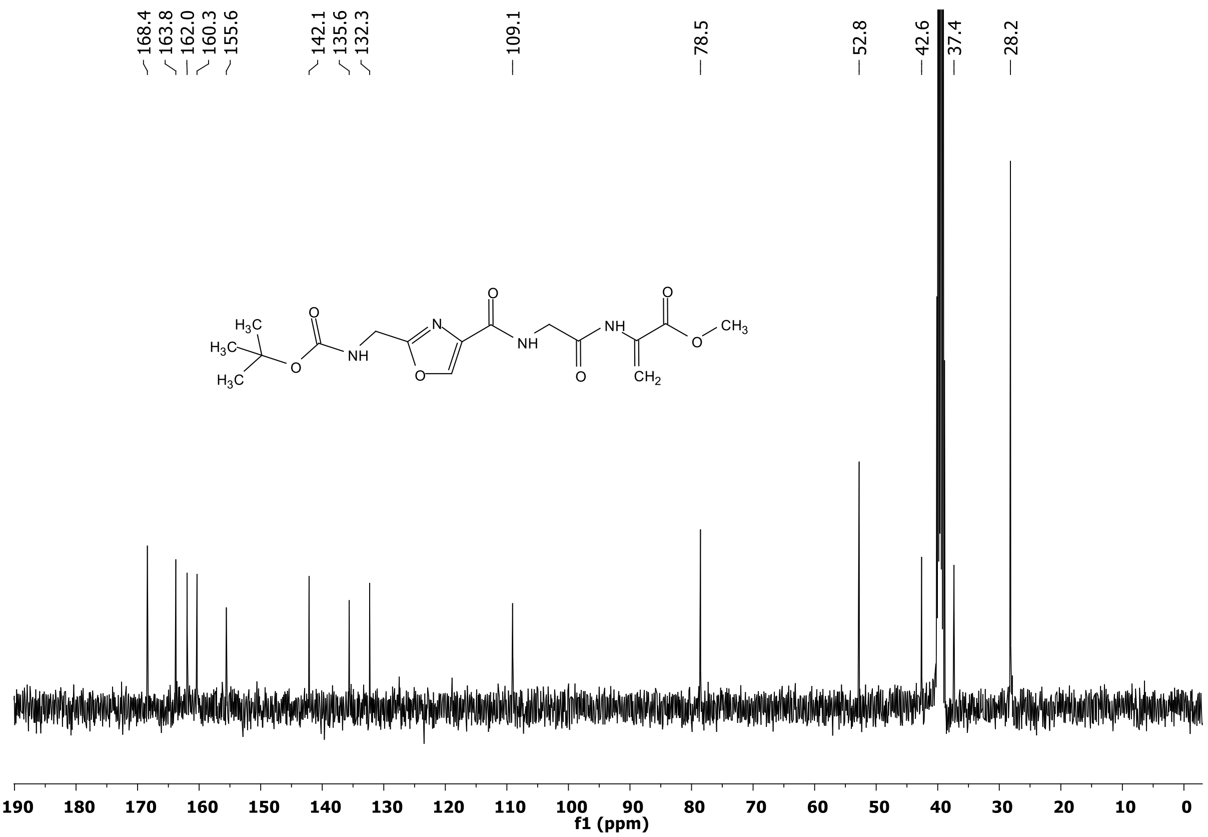
168.4
163.8
162.0
160.3
155.6
142.1
135.6
132.3
109.1
78.5
52.8
42.6
37.4
28.2
f1 (ppm)

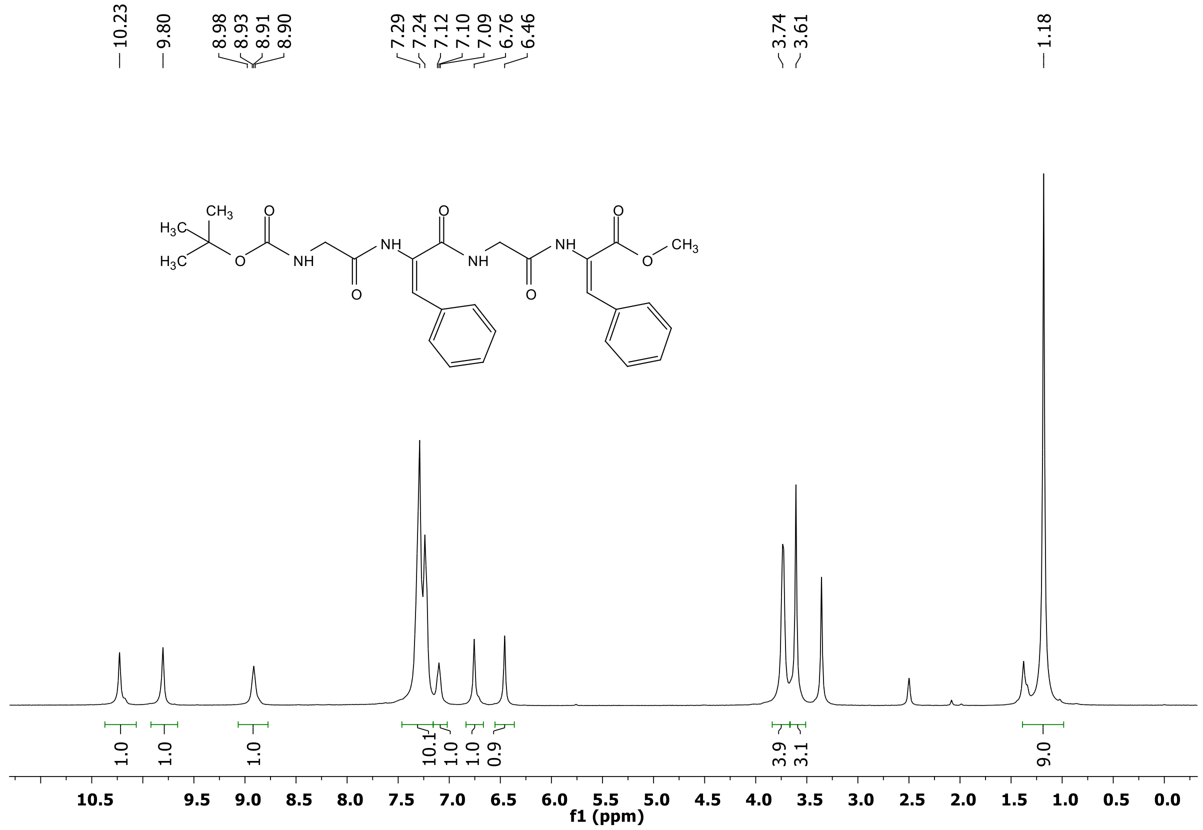
10.23
9.80
8.98
8.93
8.91
8.90
7.29
7.24
7.12
7.10
7.09
6.76
6.46
3.74
3.61
1.18
1.0
1.0
1.0
10.1
1.0
1.0
0.9
3.9
3.1
9.0
f1 (ppm)

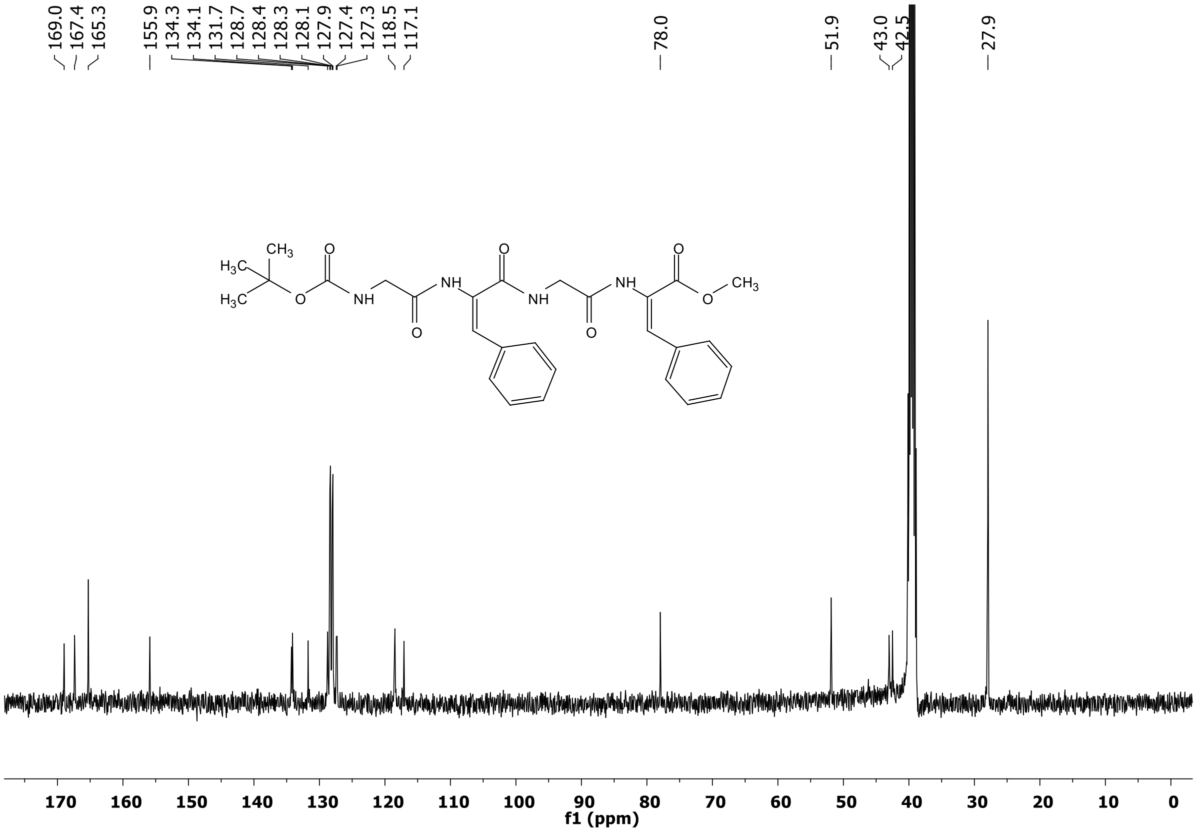
169.0
167.4
165.3
155.9
134.3
134.1
131.7
128.7
128.4
128.3
128.1
127.9
127.4
127.3
118.5
117.1
78.0
51.9
43.0
42.5
27.9
170 160 150 140 130 120 110 100 90 80 70 60 50 40 30 20 10 0
f1 (ppm)

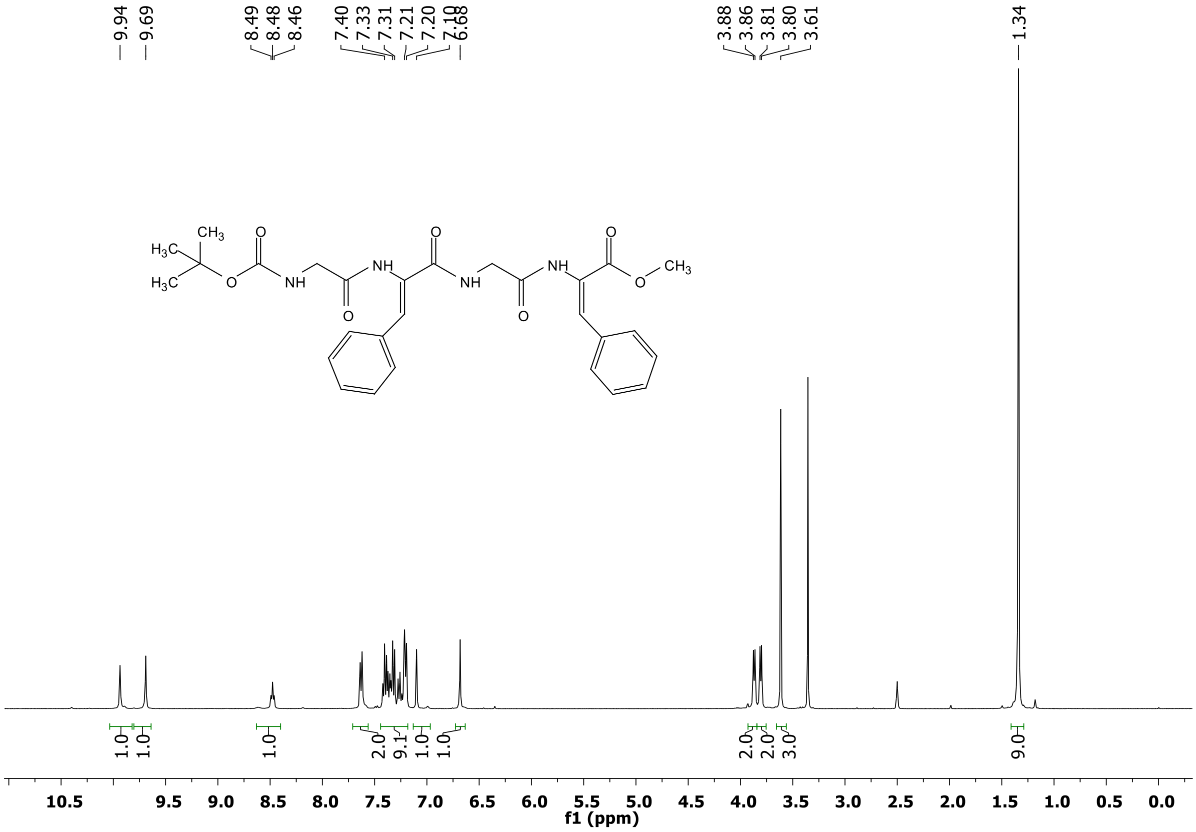
9.94
9.69
8.49
8.48
8.46
7.40
7.33
7.31
7.21
7.20
7.10
6.68
3.88
3.86
3.81
3.80
3.61
1.34
1.0 1.0 1.0 2.0 9.1 1.0 1.0 2.0 2.0 3.0 9.0
10.5 9.5 9.0 8.5 8.0 7.5 7.0 6.5 6.0 5.5 5.0 4.5 4.0 3.5 3.0 2.5 2.0 1.5 1.0 0.5 0.0
f1 (ppm)

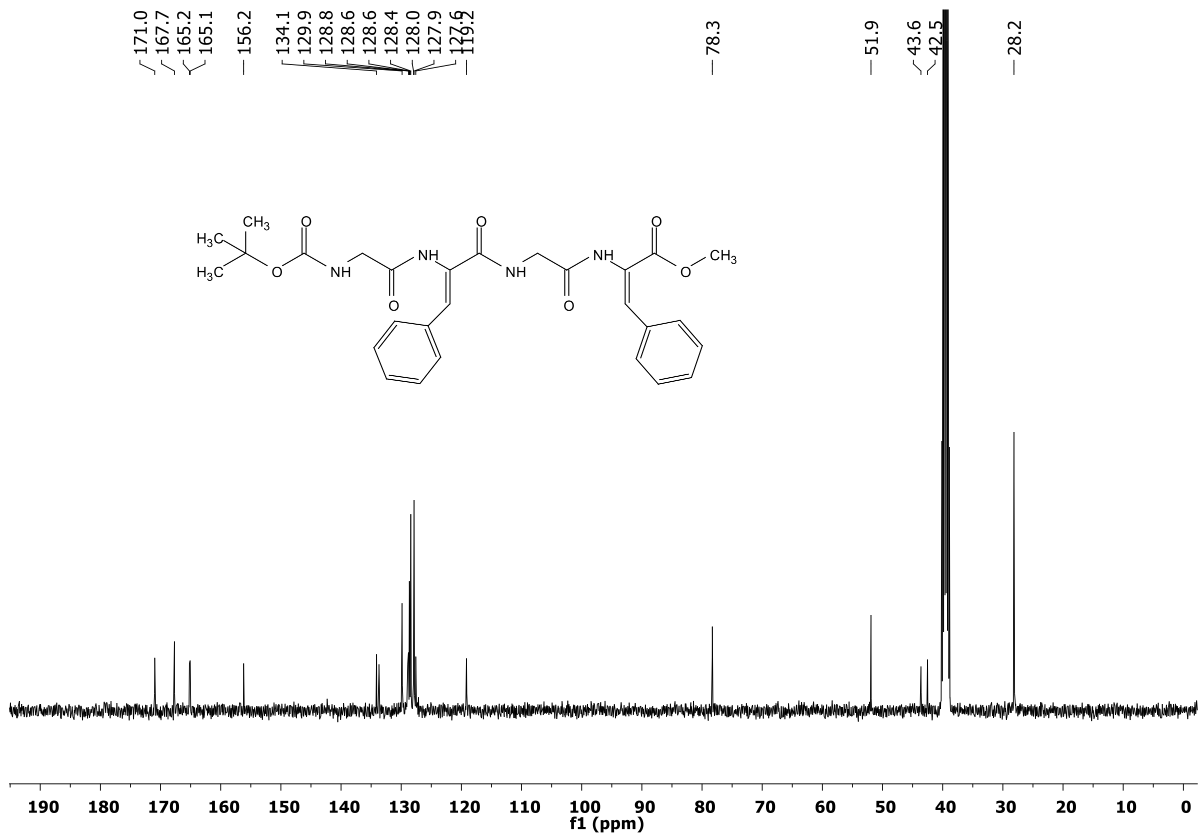

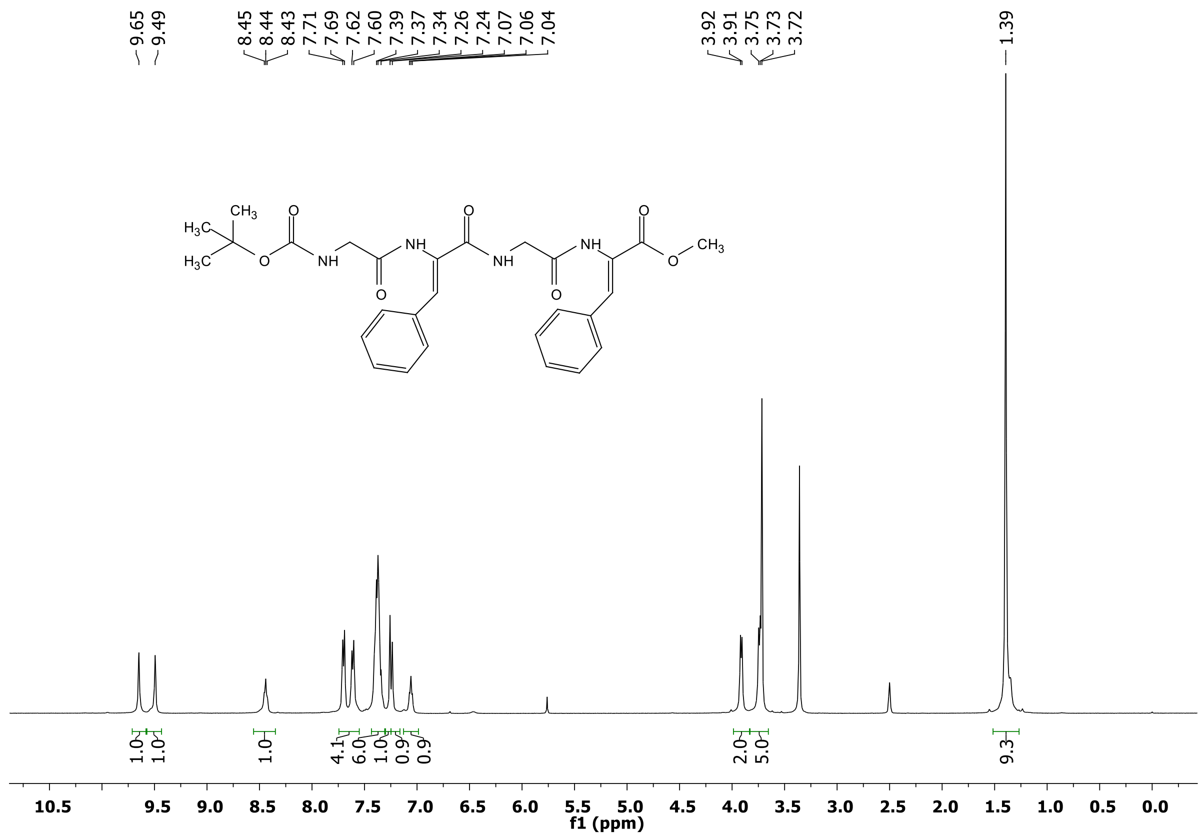

The signal derived from one carbon atom in the aromatic/vinyl region of spectrum is not visible because of signals overlapping.

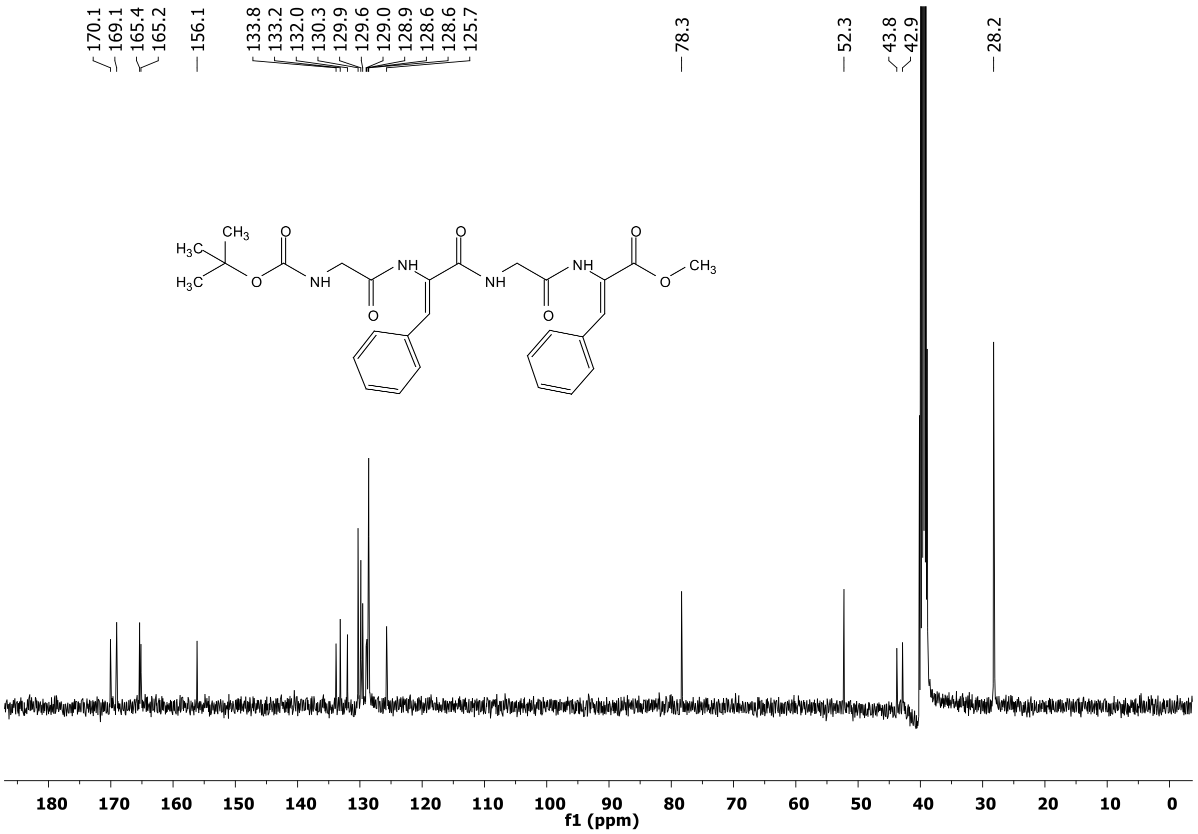
170.1
169.1
165.4
165.2
156.1
133.8
133.2
132.0
130.3
129.9
129.6
129.0
128.9
128.6
128.6
125.7
78.3
52.3
43.8
42.9
28.2
f1 (ppm)

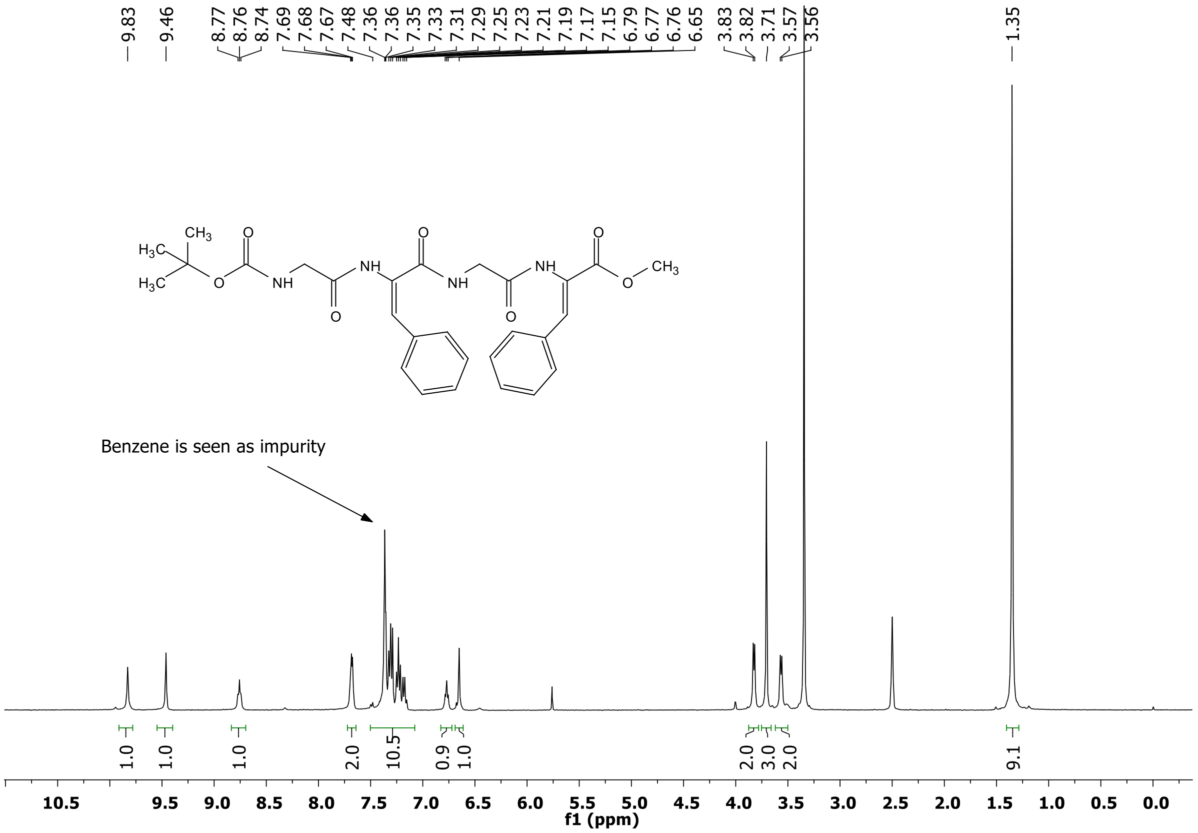
9.83
9.46
8.77
8.76
8.74
7.69
7.68
7.67
7.48
7.36
7.36
7.35
7.33
7.31
7.29
7.25
7.23
7.21
7.19
7.17
7.15
6.79
6.77
6.76
6.65
3.83
3.82
3.71
3.57
3.56
1.35
Benzene is seen as impurity
f1 (ppm)

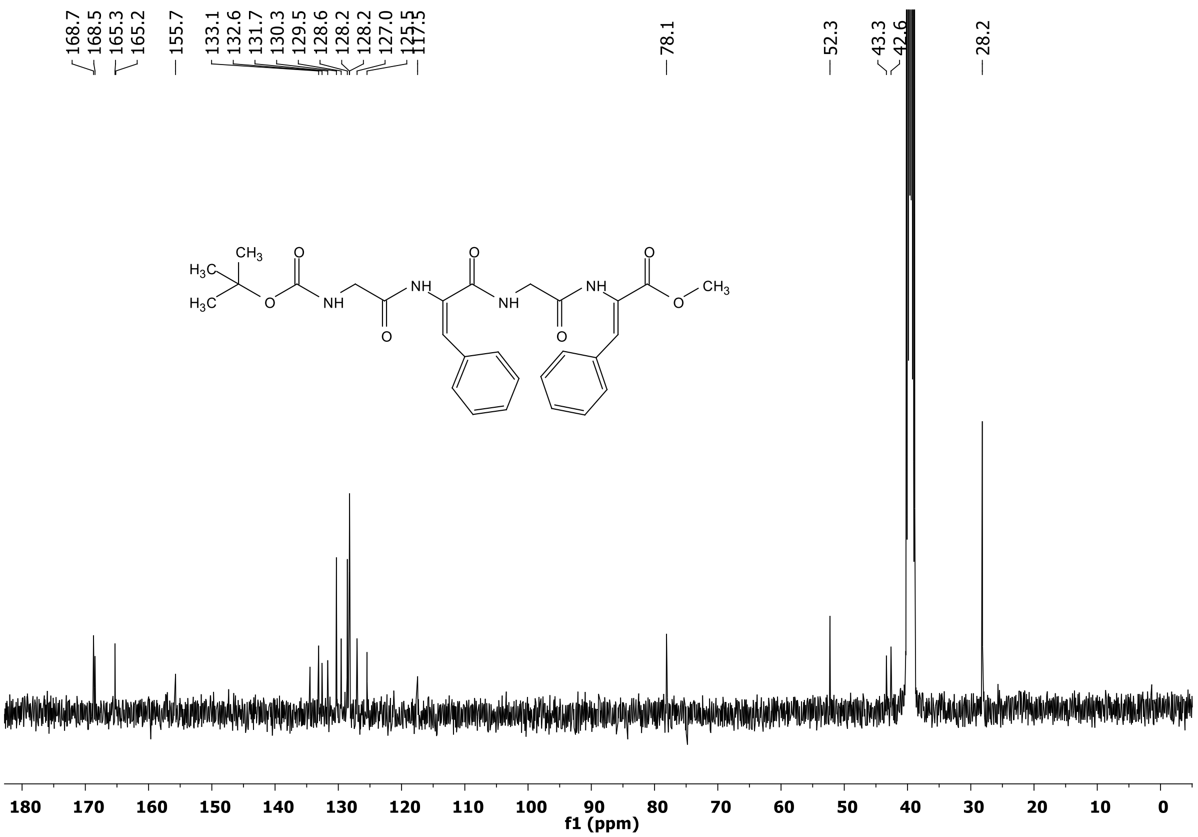
168.7
168.5
165.3
165.2
155.7
133.1
132.6
131.7
130.3
129.5
128.6
128.2
128.2
127.0
125.5
117.5
78.1
52.3
43.3
42.6
28.2
f1 (ppm)

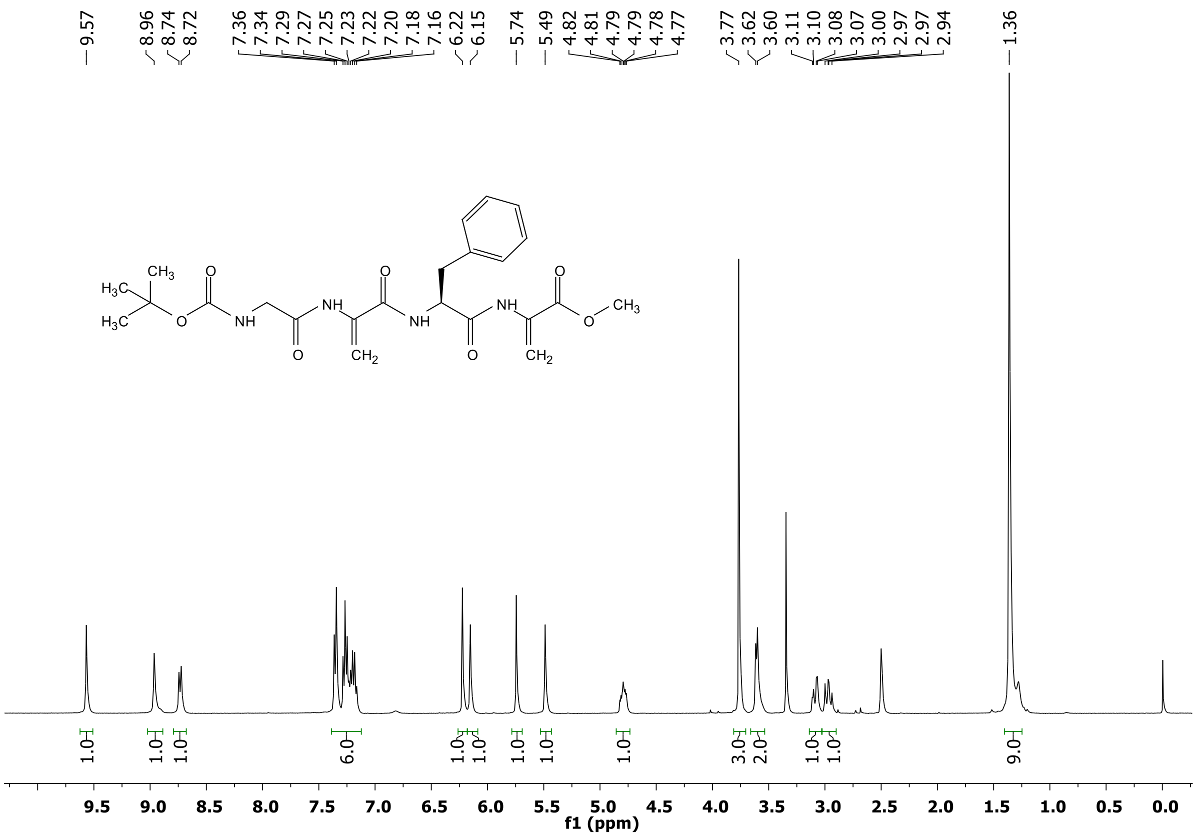
9.57
8.96
8.74
8.72
7.36
7.34
7.29
7.27
7.25
7.23
7.22
7.20
7.18
7.16
6.22
6.15
5.74
5.49
4.82
4.81
4.79
4.79
4.78
4.77
3.77
3.62
3.60
3.11
3.10
3.08
3.07
3.00
2.97
2.97
2.94
1.36
1.0
1.0
1.0
6.0
1.0
1.0
1.0
1.0
1.0
3.0
2.0
1.0
1.0
9.0
f1 (ppm)

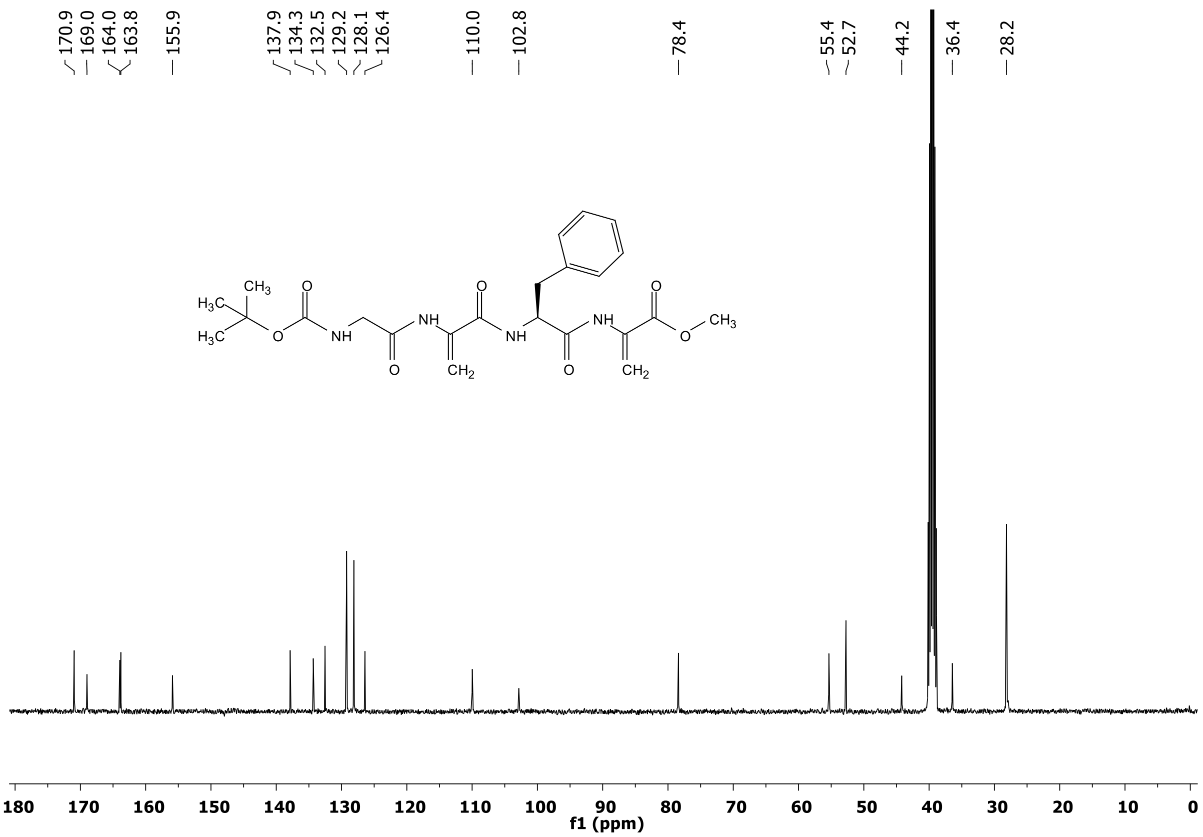

4. Crystallographic data

Table S2 Experimental details for compounds **3**, **14** and **15**.

	Compound **15**	Compound **3**	Compound **14**
Crystal data			
Chemical formula	$C_8H_9F_3N_2O_5$	$C_{16}H_{22}N_4O_7$	$C_{11}H_{16}N_2O_5$
M_r	270.17	382.37	256.26
Crystal system, space group	Triclinic, $P\bar{1}$	Orthorhombic, *Fdd*2	Monoclinic, $P2_1$
a, *b*, *c* (Å)	6.761 (2), 8.200 (3), 11.126 (2)	35.4974 (17), 21.7588 (13), 10.2566 (11)	5.1373 (5), 11.1775 (13), 11.6023 (14)
α, β, γ (°)	97.11 (2), 105.09 (2), 107.05 (3)	90, 90, 90	90, 90, 90
V (Å^3)	555.9 (3)	7922.0 (10)	666.23 (13)
Z	2	16	2
μ (mm^{-1})	0.16	0.10	0.10
Crystal size (mm)	0.1 × 0.08 × 0.03	0.3 × 0.2 × 0.1	0.15 × 0.1 × 0.05
Data collection			
Diffractometer	KM4 with Eos CCD	KM4 with Eos CCD	Xcalibur, Sapphire2
T_{min}, T_{max}	0.565, 1.000	0.879, 1.000	0.475, 1.000
No. of measured, independent and observed [$I > 2\sigma(I)$] reflections	3548, 2173, 1100	8223, 3392, 1949	4423, 2552, 1826
R_{int}	0.060	0.024	0.075
$(\sin \theta/\lambda)_{max}$ (Å^{-1})	0.617	0.617	0.616

Refinement			
$R[F^2 > 2\sigma(F^2)]$, $wR(F^2)$, S	0.115, 0.350, 1.04	0.054, 0.160, 1.04	0.096, 0.287, 1.07
No. of reflections	2173	3392	2552
No. of parameters	165	260	170
No. of restraints	0	404	1
H-atom treatment	H-atom parameters constrained	H atoms treated by a mixture of independent and constrained refinement	H atoms treated by a mixture of independent and constrained refinement
$\Delta\rho_{max}$, $\Delta\rho_{min}$ (e Å^{-3})	1.07, −0.53	0.16, −0.14	0.35, −0.33

Table S3. Selected geometric parameters (Å, º) for **3**, **14** and **15.**

**15**			
F1—C8	1.338 (10)	O3—C5	1.172 (7)
F2—C8	1.264 (8)	O4—C7	1.227 (7)
F3—C8	1.289 (8)	O5—C7	1.232 (7)
N1—C1	1.290 (8)	C1—C4	1.469 (8)
N1—C3	1.400 (7)	C2—C3	1.320 (9)
N2—C4	1.446 (7)	C2—H2	0.9300
N2—H2A	0.8900	C3—C5	1.459 (9)
N2—H2B	0.8900	C4—H4A	0.9700
N2—H2C	0.8900	C4—H4B	0.9700
O1—C1	1.357 (7)	C6—H6A	0.9600
O1—C2	1.365 (8)	C6—H6B	0.9600
O2—C5	1.326 (7)	C6—H6C	0.9600
O2—C6	1.436 (8)	C7—C8	1.498 (9)
C1—N1—C3	104.0 (5)	N2—C4—H4B	109.6
C4—N2—H2A	109.5	C1—C4—H4B	109.6
C4—N2—H2B	109.5	H4A—C4—H4B	108.1
H2A—N2—H2B	109.5	O3—C5—O2	124.8 (6)
C4—N2—H2C	109.5	O3—C5—C3	125.3 (6)
H2A—N2—H2C	109.5	O2—C5—C3	109.9 (5)
H2B—N2—H2C	109.5	O2—C6—H6A	109.5
C1—O1—C2	103.1 (5)	O2—C6—H6B	109.5
C5—O2—C6	116.9 (5)	H6A—C6—H6B	109.5
N1—C1—O1	114.6 (5)	O2—C6—H6C	109.5
N1—C1—C4	130.1 (5)	H6A—C6—H6C	109.5
O1—C1—C4	115.3 (5)	H6B—C6—H6C	109.5
C3—C2—O1	109.8 (6)	O4—C7—O5	131.0 (6)
C3—C2—H2	125.1	O4—C7—C8	114.2 (6)
O1—C2—H2	125.1	O5—C7—C8	114.8 (5)
C2—C3—N1	108.5 (6)	F2—C8—F3	112.6 (8)
C2—C3—C5	129.8 (6)	F2—C8—F1	97.2 (7)
N1—C3—C5	121.7 (5)	F3—C8—F1	99.2 (7)
N2—C4—C1	110.5 (5)	F2—C8—C7	117.5 (6)
N2—C4—H4A	109.6	F3—C8—C7	117.3 (6)
C1—C4—H4A	109.6	F1—C8—C7	109.2 (7)

C3—N1—C1—O1	1.0 (7)	C6—O2—C5—C3	179.3 (6)
C3—N1—C1—C4	179.2 (6)	C2—C3—C5—O3	−176.9 (7)
C2—O1—C1—N1	−1.3 (7)	N1—C3—C5—O3	0.3 (11)
C2—O1—C1—C4	−179.8 (6)	C2—C3—C5—O2	3.8 (10)
C1—O1—C2—C3	1.0 (8)	N1—C3—C5—O2	−179.0 (5)
O1—C2—C3—N1	−0.5 (8)	O4—C7—C8—F2	−25.1 (11)
O1—C2—C3—C5	177.0 (6)	O5—C7—C8—F2	156.2 (8)
C1—N1—C3—C2	−0.2 (7)	O4—C7—C8—F3	−164.2 (8)
C1—N1—C3—C5	−178.0 (6)	O5—C7—C8—F3	17.1 (11)
N1—C1—C4—N2	9.5 (10)	O4—C7—C8—F1	84.2 (8)
O1—C1—C4—N2	−172.3 (5)	O5—C7—C8—F1	−94.5 (7)
C6—O2—C5—O3	0.0 (10)		
**3**			
N1—C5	1.284 (6)	C1B—H1BC	0.9600
N1—C3	1.392 (9)	C2—C3	1.479 (9)
N2—C7	1.322 (6)	C3—C4	1.330 (9)
N2—C6	1.441 (6)	C4—H4	0.9300
N2—H2	0.8600	C5—C6	1.479 (8)
N3—C10	1.355 (6)	C6—H6A	0.9700
N3—C8	1.402 (5)	C6—H6B	0.9700
N3—H3	0.8600	C17—C14	1.490 (14)
O1—C2	1.274 (9)	C17—H17A	0.9600
O1—C1A	1.467 (12)	C17—H17B	0.9600
O2—C2	1.231 (10)	C17—H17C	0.9600
O2—C1B	1.437 (17)	C7—C8	1.500 (7)
O3—C5	1.359 (8)	C8—C9	1.304 (7)
O3—C4	1.365 (7)	C9—H9A	0.9300
O4—C7	1.224 (5)	C9—H9B	0.9300
N4—C13	1.321 (10)	C10—C11	1.501 (7)
N4—C11	1.420 (7)	C11—H11A	0.9700
N4—H4A	0.8600	C11—H11B	0.9700
O5—C10	1.203 (6)	C14—C16	1.500 (12)
O6—C13	1.195 (10)	C14—C15	1.508 (12)
O7—C13	1.342 (7)	C15—H15A	0.9600
O7—C14	1.468 (10)	C15—H15B	0.9600
C1A—H1AA	0.9600	C15—H15C	0.9600
C1A—H1AB	0.9600	C16—H16A	0.9600
C1A—H1AC	0.9600	C16—H16B	0.9600
C1B—H1BA	0.9600	C16—H16C	0.9600
C1B—H1BB	0.9600		
C5—N1—C3	104.7 (6)	C14—C17—H17B	109.5
C7—N2—C6	121.1 (4)	H17A—C17—H17B	109.5
C7—N2—H2	119.5	C14—C17—H17C	109.5
C6—N2—H2	119.5	H17A—C17—H17C	109.5
C10—N3—C8	127.2 (4)	H17B—C17—H17C	109.5
C10—N3—H3	116.4	O4—C7—N2	122.7 (4)
C8—N3—H3	116.4	O4—C7—C8	119.7 (4)

C2—O1—C1A	131.0 (9)	N2—C7—C8	117.6 (4)
C2—O2—C1B	134.9 (12)	C9—C8—N3	126.1 (5)
C5—O3—C4	104.9 (5)	C9—C8—C7	123.9 (4)
C13—N4—C11	123.2 (7)	N3—C8—C7	110.0 (4)
C13—N4—H4A	118.4	C8—C9—H9A	120.0
C11—N4—H4A	118.4	C8—C9—H9B	120.0
C13—O7—C14	121.8 (7)	H9A—C9—H9B	120.0
O1—C1A—H1AA	109.5	O5—C10—N3	123.1 (4)
O1—C1A—H1AB	109.5	O5—C10—C11	123.1 (4)
H1AA—C1A—H1AB	109.5	N3—C10—C11	113.7 (4)
O1—C1A—H1AC	109.5	N4—C11—C10	113.6 (5)
H1AA—C1A—H1AC	109.5	N4—C11—H11A	108.8
H1AB—C1A—H1AC	109.5	C10—C11—H11A	108.8
O2—C1B—H1BA	109.5	N4—C11—H11B	108.8
O2—C1B—H1BB	109.5	C10—C11—H11B	108.8
H1BA—C1B—H1BB	109.5	H11A—C11—H11B	107.7
O2—C1B—H1BC	109.5	O6—C13—N4	123.9 (7)
H1BA—C1B—H1BC	109.5	O6—C13—O7	125.4 (9)
H1BB—C1B—H1BC	109.5	N4—C13—O7	110.7 (8)
O2—C2—O1	126.7 (7)	O7—C14—C17	111.1 (7)
O2—C2—C3	117.9 (8)	O7—C14—C16	102.9 (8)
O1—C2—C3	115.4 (8)	C17—C14—C16	110.5 (9)
C4—C3—N1	109.4 (5)	O7—C14—C15	109.9 (7)
C4—C3—C2	127.0 (8)	C17—C14—C15	110.3 (11)
N1—C3—C2	123.6 (7)	C16—C14—C15	112.0 (8)
C3—C4—O3	107.7 (7)	C14—C15—H15A	109.5
C3—C4—H4	126.1	C14—C15—H15B	109.5
O3—C4—H4	126.1	H15A—C15—H15B	109.5
N1—C5—O3	113.2 (6)	C14—C15—H15C	109.5
N1—C5—C6	129.1 (7)	H15A—C15—H15C	109.5
O3—C5—C6	117.6 (5)	H15B—C15—H15C	109.5
N2—C6—C5	114.2 (6)	C14—C16—H16A	109.5
N2—C6—H6A	108.7	C14—C16—H16B	109.5
C5—C6—H6A	108.7	H16A—C16—H16B	109.5
N2—C6—H6B	108.7	C14—C16—H16C	109.5
C5—C6—H6B	108.7	H16A—C16—H16C	109.5
H6A—C6—H6B	107.6	H16B—C16—H16C	109.5
C14—C17—H17A	109.5		
C1B—O2—C2—O1	−8 (2)	C6—N2—C7—O4	4.0 (9)
C1B—O2—C2—C3	173.3 (17)	C6—N2—C7—C8	−175.0 (5)
C1A—O1—C2—O2	−4.7 (15)	C10—N3—C8—C9	−8.0 (11)
C1A—O1—C2—C3	174.4 (10)	C10—N3—C8—C7	172.3 (5)
C5—N1—C3—C4	−0.3 (6)	O4—C7—C8—C9	−154.9 (6)
C5—N1—C3—C2	−180.0 (5)	N2—C7—C8—C9	24.1 (9)
O2—C2—C3—C4	175.9 (7)	O4—C7—C8—N3	24.9 (7)
O1—C2—C3—C4	−3.3 (10)	N2—C7—C8—N3	−156.1 (5)
O2—C2—C3—N1	−4.5 (10)	C8—N3—C10—O5	−0.4 (10)
O1—C2—C3—N1	176.3 (6)	C8—N3—C10—C11	178.8 (6)

N1—C3—C4—O3	0.3 (7)	C13—N4—C11—C10	−101.8 (8)
C2—C3—C4—O3	180.0 (6)	O5—C10—C11—N4	−17.4 (11)
C5—O3—C4—C3	−0.2 (6)	N3—C10—C11—N4	163.5 (6)
C3—N1—C5—O3	0.2 (6)	C11—N4—C13—O6	−1.7 (10)
C3—N1—C5—C6	176.7 (5)	C11—N4—C13—O7	179.5 (5)
C4—O3—C5—N1	0.0 (6)	C14—O7—C13—O6	6.0 (10)
C4—O3—C5—C6	−176.9 (4)	C14—O7—C13—N4	−175.2 (6)
C7—N2—C6—C5	98.4 (6)	C13—O7—C14—C17	55.5 (10)
N1—C5—C6—N2	145.7 (5)	C13—O7—C14—C16	173.7 (8)
O3—C5—C6—N2	−37.9 (6)	C13—O7—C14—C15	−66.8 (10)
**14**			
O1—C1	1.353 (10)	C4—H4A	0.9700
O1—C3	1.387 (11)	C4—H4B	0.9700
O2—C5	1.238 (8)	C6—C8	1.493 (14)
O3—C5	1.349 (9)	C6—C9	1.497 (14)
O3—C6	1.493 (10)	C6—C7	1.540 (13)
O4—C10	1.171 (12)	C7—H7A	0.9600
O5—C10	1.336 (13)	C7—H7B	0.9600
O5—C11	1.456 (12)	C7—H7C	0.9600
N1—C1	1.312 (10)	C8—H8A	0.9600
N1—C2	1.397 (11)	C8—H8B	0.9600
N2—C5	1.323 (10)	C8—H8C	0.9600
N2—C4	1.460 (11)	C9—H9A	0.9600
N2—H2	0.93 (17)	C9—H9B	0.9600
C1—C4	1.478 (11)	C9—H9C	0.9600
C2—C3	1.312 (13)	C11—H11A	0.9600
C2—C10	1.505 (11)	C11—H11B	0.9600
C3—H3	0.9300	C11—H11C	0.9600
C1—O1—C3	104.6 (6)	C8—C6—C7	109.5 (10)
C5—O3—C6	120.8 (5)	C9—C6—C7	111.0 (8)
C10—O5—C11	116.0 (9)	C6—C7—H7A	109.5
C1—N1—C2	102.7 (7)	C6—C7—H7B	109.5
C5—N2—C4	119.9 (6)	H7A—C7—H7B	109.5
C5—N2—H2	115.4	C6—C7—H7C	109.5
C4—N2—H2	122.1	H7A—C7—H7C	109.5
N1—C1—O1	114.0 (7)	H7B—C7—H7C	109.5
N1—C1—C4	127.0 (7)	C6—C8—H8A	109.5
O1—C1—C4	119.0 (7)	C6—C8—H8B	109.5
C3—C2—N1	111.6 (7)	H8A—C8—H8B	109.5
C3—C2—C10	126.1 (8)	C6—C8—H8C	109.5
N1—C2—C10	122.3 (7)	H8A—C8—H8C	109.5
C2—C3—O1	107.1 (7)	H8B—C8—H8C	109.5
C2—C3—H3	126.4	C6—C9—H9A	109.5
O1—C3—H3	126.4	C6—C9—H9B	109.5
N2—C4—C1	111.5 (6)	H9A—C9—H9B	109.5
N2—C4—H4A	109.3	C6—C9—H9C	109.5
C1—C4—H4A	109.3	H9A—C9—H9C	109.5

N2—C4—H4B	109.3	H9B—C9—H9C	109.5
C1—C4—H4B	109.3	O4—C10—O5	124.8 (9)
H4A—C4—H4B	108.0	O4—C10—C2	124.5 (9)
O2—C5—N2	124.6 (7)	O5—C10—C2	110.7 (7)
O2—C5—O3	124.7 (7)	O5—C11—H11A	109.5
N2—C5—O3	110.7 (6)	O5—C11—H11B	109.5
O3—C6—C8	111.1 (7)	H11A—C11—H11B	109.5
O3—C6—C9	110.4 (8)	O5—C11—H11C	109.5
C8—C6—C9	112.1 (7)	H11A—C11—H11C	109.5
O3—C6—C7	102.3 (6)	H11B—C11—H11C	109.5
C2—N1—C1—O1	−0.3 (8)	C4—N2—C5—O3	−180.0 (7)
C2—N1—C1—C4	180.0 (7)	C6—O3—C5—O2	−5.8 (12)
C3—O1—C1—N1	0.7 (9)	C6—O3—C5—N2	173.2 (7)
C3—O1—C1—C4	−179.5 (7)	C5—O3—C6—C8	66.3 (11)
C1—N1—C2—C3	−0.3 (9)	C5—O3—C6—C9	−58.6 (10)
C1—N1—C2—C10	−178.7 (7)	C5—O3—C6—C7	−176.9 (9)
N1—C2—C3—O1	0.8 (10)	C11—O5—C10—O4	−1.3 (14)
C10—C2—C3—O1	179.0 (7)	C11—O5—C10—C2	−179.3 (8)
C1—O1—C3—C2	−0.9 (9)	C3—C2—C10—O4	−4.0 (14)
C5—N2—C4—C1	56.6 (10)	N1—C2—C10—O4	174.0 (8)
N1—C1—C4—N2	−141.1 (8)	C3—C2—C10—O5	173.9 (10)
O1—C1—C4—N2	39.2 (10)	N1—C2—C10—O5	−8.0 (10)
C4—N2—C5—O2	−1.0 (12)		

Table S4. Selected hydrogen-bond parameters for **14** and **15.**

D—H···*A*	*D*—H (Å)	H···*A* (Å)	*D*···*A* (Å)	*D*—H···*A* (°)
**15**				
N2—H2···O5^{i}	0.86	2.02	2.826 (5)	155.1
N3—H3···O4^{ii}	0.86	2.12	2.955 (5)	164.7
N4—H4A···N1^{iii}	0.86	2.40	3.088 (10)	137.6
C1A—H1AB···O4^{iv}	0.96	2.55	3.394 (17)	146.5
C1B—H1BA···N3^{v}	0.96	2.61	3.44 (2)	145.5
C4—H4···O6^{vi}	0.93	2.35	3.116 (11)	139.2
C6—H6B···O7^{v}	0.97	2.58	3.538 (9)	171.2
C17—H17C···O6	0.96	2.34	2.955 (12)	121.0
C9—H9A···O5	0.93	2.25	2.841 (6)	120.9
C11—H11B···O4^{ii}	0.97	2.48	3.124 (7)	123.8
**14**				
N2—H2···O2^{vii}	0.93	2.17	2.944 (7)	140.3

Symmetry code(s): (i) −*x*+3/4, *y*+1/4, *z*−1/4; (ii) −*x*+1/2, −*y*+1/2, *z*; (iii) *x*, *y*−1/2, *z*+1/2; (iv) −*x*+1/2, −*y*+1, *z*+1/2; (v) *x*, *y*+1/2, *z*−1/2; (vi) *x*, *y*+1/2, *z*+1/2; (vii) *x*+1, *y*, *z*.
